# The transmission potential of Rift Valley fever virus among livestock in the Netherlands: a modelling study

**DOI:** 10.1186/1297-9716-44-58

**Published:** 2013-07-22

**Authors:** Egil AJ Fischer, Gert-Jan Boender, Gonnie Nodelijk, Aline A de Koeijer, Herman JW van Roermund

**Affiliations:** 1 Central Veterinary Institute, Part of Wageningen UR, Lelystad, The Netherlands

## Abstract

**Abstracts:**

Rift Valley fever virus (RVFV) is a zoonotic vector-borne infection and causes a potentially severe disease. Many mammals are susceptible to infection including important livestock species. Although currently confined to Africa and the near-East, this disease causes concern in countries in temperate climates where both hosts and potential vectors are present, such as the Netherlands. Currently, an assessment of the probability of an outbreak occurring in this country is missing. To evaluate the transmission potential of RVFV, a mathematical model was developed and used to determine the initial growth and the Floquet ratio, which are indicators of the probability of an outbreak and of persistence in a periodic changing environment caused by seasonality. We show that several areas of the Netherlands have a high transmission potential and risk of persistence of the infection. Counter-intuitively, these are the sparsely populated livestock areas, due to the high vector-host ratios in these areas. *Culex pipiens* s.l. is found to be the main driver of the spread and persistence, because it is by far the most abundant mosquito. Our investigation underscores the importance to determine the vector competence of this mosquito species for RVFV and its host preference.

## Introduction

Rift Valley fever virus (RVFV; Bunyaviridae: Phlebovirus) was first isolated during an outbreak in the 1930’s in the Rift Valley of Kenya [1]. Between 1930 and 1977 outbreaks of RVF were limited to sub-Saharan Africa [2]. In 1977 the first documented outbreak north of the Sahara occurred in Egypt, and since that time RVFV has been found in Madagascar and smaller islands of the coast of mainland Africa [2]. In 2000 the first outbreak occurred outside Africa on the Arabian Peninsula in Saudi Arabia and Yemen [3]. The increasing known area of distribution and an outbreak out of Africa feeds the fear of an expansion of the area affected by RVF and especially into the direction of the Middle East and Europe [2].

Many mammalian species are susceptible to infection with RVFV, including livestock such as cattle, goat, sheep and camels [1,4], but also wildlife such as giraffe and African buffalo [5,6]. RVFV infection in susceptible livestock animals causes abortion in pregnant animals and high mortality rates of new-borns. In older animals, infection is generally mild or asymptomatic. Birds and reptiles are refractory, and important livestock species such as pigs [7], horses and other equines are resistant to the infection [4,8]. Susceptibility of deer-species (either European, Asian or American) is unknown to date.

Humans can become infected with RVFV following contact with infected animals or animal products, and although less likely as a result of a mosquito bite. RVFV infection in humans is generally mild or unapparent, but occasional severe and potentially fatal complications occur [2,4].

RVFV is a vector-borne infection primarily transmitted by mosquitoes of many different species. These include *Culex pipiens* sensu lato (L.) and *Aedes vexans* (Meigen), which are both present in the Netherlands [8]. One African vector species, *Aedes lineatopennis* (Ludlow), has been found to transmit the virus to their eggs [9]. Based on this knowledge, vectors of the genus *Aedes* are thought to transmit the virus to their eggs (vertical transmission) [8,10]. Other genera are thought not to transmit the virus vertically. Mechanical transmission of the virus by other insects, such as stable flies, has been observed in experiments [11].

The infection has entered and established itself in regions previously free of RVFV [12]. The presence of vectors and hosts does not automatically imply that an outbreak will occur after introduction. Other epidemiological, ecological and environmental factors, such as temperature, host species composition and habitat overlap, determine the probability of an outbreak. To assess the probability of an outbreak and persistence in a country in the temperate zone we have chosen the Netherlands as case. The Netherlands is a country with high and low density livestock areas and potentially competent vectors are present [13-15]. An outbreak of RVF in the Netherlands would lead to massive economic losses, and poses a risk for public health and animal welfare [16]. Therefore the transmission potential of RVFV needs to be evaluated under the climatological and epidemiological characteristics of this country.

Previous models of Rift Valley fever epidemiology either reproduced a spatial epidemic using a network model [17], or incorporated detailed information on the different stages of the vector [10], or incorporated transmission to humans [18]. These models have shown the importance of decreasing the vector life span to reduce the potential of a major outbreak. These models do not, however, include seasonality of vector populations or changing parameters values due to temperature fluctuations. These effects are of importance to determine risk of persistence in areas with clear temporal fluctuations, such as the Netherlands.

For our purpose, a new mathematical model was developed to study the probability of a RVFV outbreak, and the probability of persistence of the infection during consecutive years. First, we apply the model to create risk maps of the Netherlands showing high risk areas for a RVFV outbreak and for persistence of RVFV in livestock. For these maps we consider host species to be cattle, sheep and goats, and consider vector species to be *Aedes vexans vexans* and *Culex pipiens* sensu lato as vectors [8]. Finally, we conducted an uncertainty analysis of the input parameters, which yielded knowledge about influential input parameters and data gaps, which can help focus future research and improve the accuracy of the model predictions.

## Material and methods

### The mathematical model: general overview

**Figure 1:**
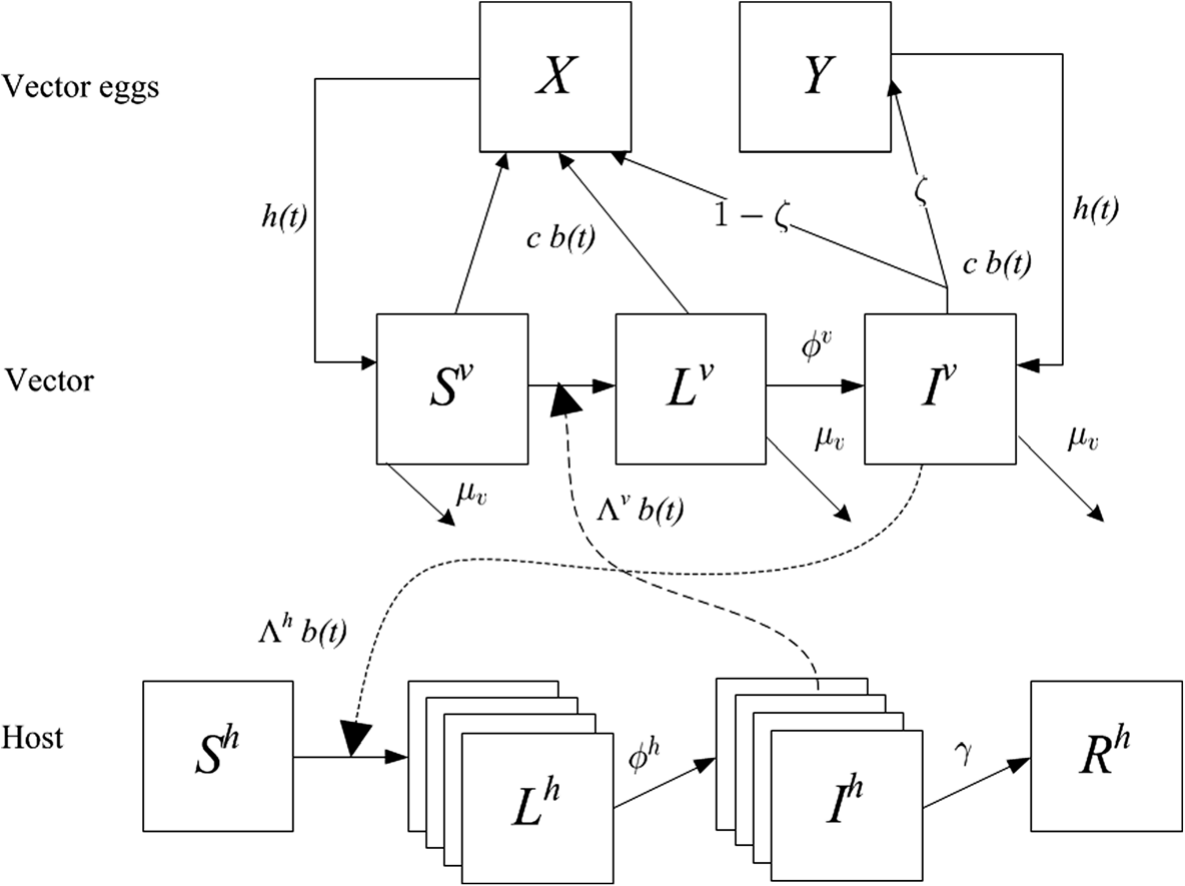
**Schematic flowchart of the model.** The boxes (compartments) depict the variables: *X* = uninfected vector eggs, *Y* = infected vector eggs, *S*^*v*^ = susceptible vectors, *L*^*v*^ = vectors in the extrinsic incubation period, *I*^*v*^ = infectious vectors, *S*^*h*^ = susceptible hosts, *L*^*h*^ = latently infected hosts, *I*^*h*^ = infectious hosts, *R* = recovered and immune hosts. The solid-line arrows depict the flow out of and into compartments. Dashed line depict the influence of infectious vectors and infectious hosts on the flow from susceptible to latent infection and extrinsic incubation period. Next to the arrows are the parameters determining that flow or influence: *h(t)* = hatching rate at time *t*, *b(t)* = biting rate at time *t*, *c* = egg batch size, ζ = per egg vertical transmission probability, *ϕ*^v^ = transition rate of vector from extrinsic incubation period to infectious state, *μ*^*v*^ = mortality rate of vector, Λ^*v*^ = per bite transmission from one infected individual of host to a susceptible vector, Λ^*h*^ = the host specific per bite transmission from a vector to a host, *ϕ*^h^ = transition rate of host from the latent state to the infectious state, *γ* = recovery rate of host.

The transmission potential of RVFV in the Netherlands is assessed using a deterministic mathematical model. Figure 1 shows the flowchart of the model. The parameters and descriptions are given in Table 1. Detailed information on the equations and model quantification is given in the Additional file 1. This model describes the local spread of the infection in a predefined small area in which all hosts and vectors mix homogeneously. In this study 5 by 5 kilometre area grids were used, based on the highest possible resolution for modelled mosquito abundances [19].

**Table 1:** Parameters and their definitions

**Parameter**	**Definition**
*1/φ*^*h*^	Average latent period of host
*γ*^h^_*_k	Mean infectious period of host
*(γ*^*h*^*)*^2^_*_k	Variance infectious period of host
*ψ*^*h*^	Host mortality
*1/μ*^*v*^_*Aedes*_*(T) = a*_*0*_*- a*_*1*_*T*	Longevity of *Ae. vexans* as function of temperature *T*
*1/μ*^*v*^_*Culex*_*(T) = a*_*0*_*- a*_*1*_*T*	Longevity of *Cx. pipiens* as function of temperature *T*
*d*^*v*^_*Culex*_	Increased mortality of infected *Cx. pipiens*
*b(T)*_*mosquitoes*_ *= b*_*slope*_*(T- b*_*min*_*)*	Biting rate of mosquito species as function of temperature *T*
*1/φ*^*v*^_*Aedes*_*(T) = 1/( φ*_*max*_*- φ*_*slope*_*T)*	Extrinsic incubation period *Ae. vexans* as function of temperature *T*. The slope is equal for *Ae. vexans* and *Cx. pipiens*.
*1/φ*^*v*^_*Culex*_*(T) = 1/( φ*_*max*_*- φ*_*slope*_*T)*	Extrinsic incubation period *Cx. pipiens* as function of temperature *T*. The slope is equal for *Ae. vexans* and *Cx. pipiens*
*ζ*	Vertical transmission *Ae. vexans*
*β*_*Aedes*_	Host to *Ae. vexans* transmission probability
*β*_*Culex*_	Host to *Cx. pipiens* transmission probability
*α*_*Aedes*_	*Ae. vexans* to host transmission probability
*α*_*Culex*_	*Cx. pipiens* to host transmission probability
*π*_*Aedes, j*_	Relative preference of *Ae. vexans* for a host species *j*
*π*_*Culex, j*_	Relative preference of *Cx. pipiens* for a host species *j*

We assume constant host population sizes and no effect of temperature on host related parameters. In contrast, vector-related parameters are temperature dependent based on average daily (24 h) temperatures in The Netherlands between 1971–2000 [20]. Furthermore, vector populations fluctuate during the vector season. The vector season is the period in which vectors are active between 21^st^ of April and 23^rd^ of October, given the temperature threshold for biting of 9.6 °C [21].

Activity and survival of mosquitoes during winter months and especially how that affects the virus, is poorly understood. Therefore, in our model we assume a period of stasis during winter, i.e. the number of susceptible and infected vectors and the number of susceptible, infected and recovered hosts at the beginning of the vector season is equal to the situation at the end of the previous vector season. This implies that the infection cannot die out during the winter in our model. The rationale behind this assumption is that, without an outbreak, the overwintering strategies of vectors and the virus cannot be determined for RVFV in temperate countries like the Netherlands in which an outbreak has never occurred. For instance, the bluetongue virus overwintered in the country unexpectedly, and it is not clear how [22]. Virus had overwintered and the epidemic reactivated, when the conditions were favourable again [22]. A similar pattern is possible for RVFV.

For convenience we assumed stasis of the host as well. To test the effect of stasis of the host we performed a sensitivity analysis, to evaluate its impact (see Additional file 1). We found that a reappearing epidemic after a stasis period, very quickly returns to the pattern of a continued epidemic (Additional file 1: Figure S3).

### Host species

The main host species are domestic cattle, sheep and goat. Pigs and equines are resistant to natural infection. Rodents are not taken into account, because they are assumed to play a negligible role in the epidemiology [23]. Also humans are assumed not to add to the epidemiology. Deer are not taken into account for the creation of risk maps, because it is unknown whether these animals are susceptible to RVFV.

The abundances of livestock hosts per 5 × 5 km grid in the Netherlands were acquired from a central database, which contains the registered number of livestock per holding in the Netherlands. The location of the animals was assumed to be the location of the farm address. This database is maintained by and opened to our use by “Dienst Regelingen” of the Ministry of Economic Affairs, Agriculture and Innovation (currently the Ministry of Economic Affairs) to determine cattle, goat and sheep density.

### Vector species

**Figure 2:**
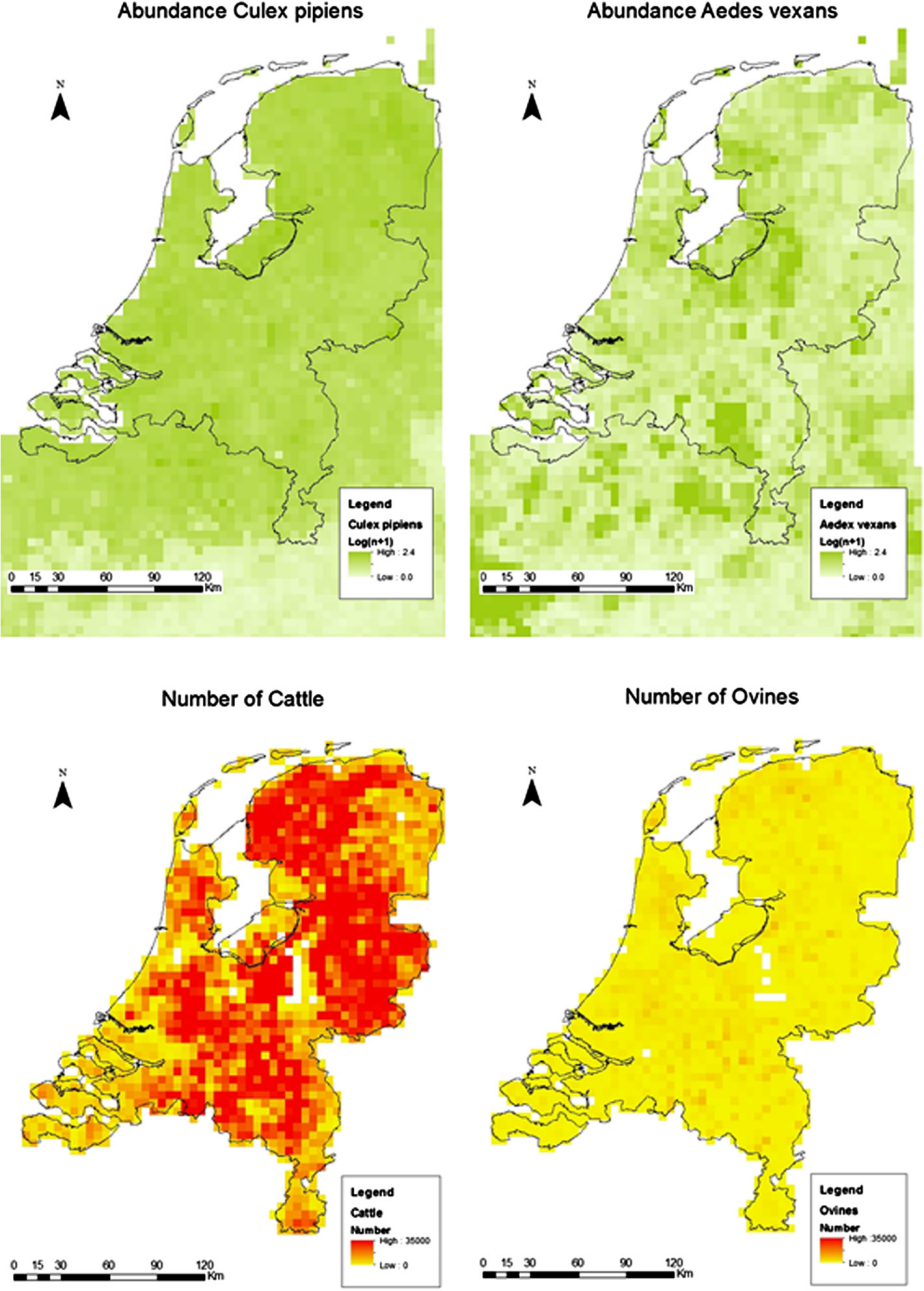
**Vector abundance of mosquito species (in Log**_**10**_**[n + 1]) and livestock abundance, per 5 by 5 km area in the Netherlands.** Vector, *Cx. pipiens s.l.* (L.) and *Ae. vexans* (Meigen), abundances are extrapolated values from Belgian data based on spatial variables [19]. Cattle and sheep & goat abundances (lower maps) are calculated from the per holding number of animals and the location of the farm addresses.

Two mosquito vector species - *Aedes vexans vexans* (Meigen), and *Culex pipiens* (L.) sensu lato - were indicated as potential vectors in the Netherlands [8]. In the Netherlands *Cx. pipiens* subspecies *pipiens* and subspecies *molestus* (Forskål) are found as well as hybrids, these three types are meant with *Cx. pipiens* sensu lato, with sensu stricto we depict *Cx*. *pipiens* subspecies *pipiens*. Mosquito abundances of *Ae. vexans* and *Cx. pipiens* s.l. (Figure 2) were determined by extrapolation from Belgian data [19]. We refer to Additional file 1 and Ducheyne et al. [19] for more details on the methods used.

The Dutch vector species are part of a species complex: *Ae. vexans vexans* and *Cx. pipiens* s.l. *Ae. vexans vexans* is the European sub-species of *Ae. vexans.* The Arabian sub-species *Ae. vexans arabiensis* (Patton) was a major vector in the Saudi-Arabian and West-African outbreaks [24-26]. We assume that the Dutch *Ae. vexans vexans* has the same vector competence as *Ae. vexans arabiensis*. We will denote this species by *Ae. vexans*. The situation for *Cx. pipiens* s.l. is more complex. The main two sub-species *Cx. pipiens* sensu stricto and *Cx. pipiens molestus* differ in host-preference. *Cx. pipiens* s.s*.* is strictly ornithophilic and does not bite on mammals, while *Cx. pipiens molestus* is opportunistic and bites on both birds and mammals. The sub-species hybridize to form populations with intermediate preferences. For the Netherlands it is unknown which species or hybrids are present (pers. com. Dr E.J. Scholte). In this study we assume that *Cx. pipiens* s.l. is purely biting on mammals as a worst case assumption, and denote the species simply by *Cx. pipiens*.

### The mathematical model: description

#### Host

Host are categorized into four states: susceptible (*S*^*h*^), latent (*L*^*h*^), infectious (*I*^*h*^) and recovered (*R*^*h*^). Host are born susceptible, and will enter the latent state after infection. After the latent state animals become infectious and after clearance of the infection will become recovered. Animals remain in the recovered class until death [4].

The force of infection is the per capita rate at which hosts are infected, thus transit from the susceptible (*S*^*h*^) to the latent state (*L*^*h*^). This rate is given by ∑i=1mΛijhbit1/Njh⋅Iiv, which sums the infection pressure from all infected vectors of *m* vector species. This summation includes the product of the numbers of infectious vectors *I*^*v*^_*i*_, the number of hosts $N_{j}^{h}$, the biting rate *b*_*i*_*(t)* of vector *i* and the term $\Lambda_{\mathrm{ij}}^{h}$. The term $\Lambda_{\mathrm{ij}}^{h}$ is the *host specific per bite transmission* from vector *i* to host *j*, which is defined as the fraction of successful transmission events from one infected vector of species *i* to a host of species *j* per bite.

(1) $$\Lambda_{\mathrm{ij}}^{h}=\alpha_{\mathrm{ij}}\cdot \frac{\pi_{\mathrm{ij}}N_{j}^{h}}{\sum_{k=1}^{n}\pi_{\mathrm{ik}}N_{k}^{h}}$$

Here, *α*_*ij*_ is the transmission probability per bite from vector species *i* to host species *j*, which is multiplied by the probability of a vector of species *i* biting a host of species *j*. The probability of biting a host of species *j* is calculated by multiplication of the preference *π*_*ij*_ for host *j* by vector species *i* with the number of hosts of species *j* (*N*^*h*^_*j*_), divided by the sum of all preferences times host population sizes. The biting rate (number of bites per vector per day) is not affected by the number of hosts nor is the composition of hosts of influence on the biting rate of an individual vector. After infection hosts are in the latent class (*L*^*h*^) with average length 1/$\varphi_{j}^{h}$ days. Thereafter, hosts have a gamma-distributed infectious period of on average *k* * $\gamma_{j}^{h}$ days. From the infectious state the hosts enter the recovered state. The hosts remain in this state until they die. Hosts die at rate $\mu_{j}^{h}$, and are replaced by birth of new susceptible animals.

#### Vector

Insect populations are highly variable during a year and therefore the vector population sizes are explicitly modelled. The vector population size *N*^*v*^_*i*_ depends on the number of new adult vectors entering the population *h*_*i*_*(t)* and the mortality of vectors *μ*^*v*^_*i*_*(t)*. For *Cx. pipiens* s.l. the mortality rate increases with a factor *d*_*i*_^*v*^ if the vector is infected by RVFV [27].

Vectors have a latent state (in entomology called the extrinsic incubation period) in which they cannot infect hosts, and which is followed by an infectious state in which they can infect hosts. These infectious vectors remain infectious until death.

The total force of infection on vectors is determined by the summation of forces of infection by different host species. This calculation includes the numbers of infectious hosts *I*^*h*^_*j*_, the biting rates *b*_*i*_*(t)* of vector *i* and the factors $\Lambda_{\mathrm{ij}}^{v}$. These factors $\Lambda_{\mathrm{ij}}^{v}$ are the probabilities of a random vector of species *i* biting an infected individual of host *j* and resulting in transmission of the virus to the vector:

(2) $$\Lambda_{\mathrm{ij}}^{v}=\beta_{\mathrm{ij}}\cdot \frac{\pi_{\mathrm{ij}}\cdot N_{j}^{h}}{\sum_{k=1}^{n}\pi_{\mathrm{ik}}N_{k}^{h}}\cdot \frac{1}{N_{j}^{h}}=\beta_{\mathrm{ij}}\cdot \frac{\pi_{\mathrm{ij}}}{\sum_{k=1}^{n}\pi_{\mathrm{ik}}N_{k}^{h}}\cdot$$

Here, *β*_*ij*_ is the transmission probability per bite of susceptible vector of type *i* on an infectious host of type *j*, which is multiplied by the probability that a bitten host of species *j* is bitten by vector species *i* (equal to Equation 1) and the probability of biting the one infectious host is given by dividing by the host population size: $1/N_{j}^{h}$.

Mosquitoes of the genus *Aedes* are thought to transmit the virus from adult to egg. We developed a simple model to mimic the influx and outflow of infected eggs for *Ae. vexans*. The rate at which one female produces eggs, is determined by the biting rate *b*_*i*_*(t)* and the batch size *c*_*i*_. The batch size *c*_*i*_ is determined in the model such that the vector population size remains equal between years (but fluctuates within a year). A fraction *ζ*_*i*_ of eggs of infectious vertical transmitting vectors will become infected. After hatching and passing through larval states, these eggs develop into infectious female vectors. Infected eggs form an extra infected state *Y*_*i*_ (see Figure 1). The number of hatching eggs is equal to the influx of new female adults *h*_*i*_*(t)*. This means that we only consider those eggs that will survive, and the larvae and pupae will subsequently survive until emerging as adults. The rate of producing eggs, *c*_*i*_*b*_*i*_*(t)*, is, thus, the number of eggs laid by a female that will survive at least until becoming an adult female.

The biting rate *b*_*i*_*(t)*, mortality rate $\mu_{i}^{v}\left(t\right)$ and rate of transition from the extrinsic incubation period $\varphi_{i}^{v}\left(t\right)$ change with time *t* due to the temperature dependence of these parameters. The average 24 h temperature is described by a sine function (see Additional file 1: Figure S1). Hatching parameter *h*_*i*_*(t)* is the number of eggs hatching at a certain moment in time and was estimated such that the population size equalled the observed seasonal pattern (see Additional file 1).

#### Initial epidemic growth rate and floquet ratio, R_T_

RVFV is not present in the Netherlands, hence the country is in the so-called disease-free equilibrium. An equilibrium can either be stable, which means that after a disturbance, the system returns to the original state. In our situation, this means that after introduction of an infected vector or host, the transmission cycle is self-limiting, i.e. no new infections occur even if enough susceptible hosts are present, and returns to the situation without the infection. The other option is that the disease-free equilibrium is unstable, meaning that a disturbance (i.e. introduction of an infected vector or host) will cause a major outbreak, which is only limited by a depletion of hosts.

The stability of the disease-free equilibrium is often determined by the well-known basic reproduction number, *R*_*0*_. The basic reproduction number, *R*_*0*_, is defined as the expected number of secondary cases caused by one primary case in a totally susceptible population [28]. However, the basic reproduction number *R*_*0*_ is difficult to determine for a seasonal system and if done, simplifying assumptions have to be made which are not valid for our purposes [28]. Therefore, we chose to use another method, the newly defined Floquet ratio, *R*_*T*_[29]. The Floquet ratio, *R*_*T*_, is the expected number of cases caused by a primary case after one complete cycle of seasons [29].

We will investigate the stability of the disease-free equilibrium at two time scales. Firstly, the initial epidemic growth rate gives a measure for growth of the number of infected hosts and vectors at the moment of introduction. We will use the initial epidemic growth rate, *r,* which can be calculated for each moment in time (as opposed to *R*_*T*_ which is only applicable to the whole cycle of seasons). The initial growth rate *r* is easily derived from the model equations that are used to calculate *R*_*T*_ as well. The initial epidemic growth rate *r* can be calculated by describing the increase in the number infections by the transmission matrix **T** and the decrease (by death and recovery) by matrix **D** (see Additional file 1). If the real part of the largest eigenvalue of this matrix (**T-D**) in the disease-free equilibrium is larger than zero using the Routh-Hurwitz criteria [30], the disease-free equilibrium is unstable and a major outbreak can occur. The initial epidemic growth rate *r* will be transformed to *e*^*r*^, so that it has the same threshold property as the Floquet ratio *R*_*T*_ (i.e. *e*^*r*^ > 1).

Secondly, we will investigate the long term epidemic growth which is the average growth in number of infected hosts and vectors over multiple years. Therefore, we have to deal with annually recurring patterns (seasonality). The recently suggested Floquet ratio *R*_*T*_[29] uses Floquet theory in analysing the long term multi-annual stability of a dynamic system. Application of Floquet theory to the field of epidemiology has been proposed previously [31], but has not been used frequently by a lack of easy applicable methods [32] or by simplification requirements of the model [29]. The algorithm for *R*_*T*_ differentiates between the short term periodic changes and long term changes in numbers of infecteds [29]. In this algorithm the matrix (**T-D**) is expanded into Fourier series, which are used to determine the Floquet ratio. If the Floquet ratio exceeds 1 the disease-free equilibrium is unstable, and the infection is expected to persist. The exact mathematics of the algorithm used to determine the Floquet ratio are published by Boender et al. [29].

### Risk maps

Areas differ in host density and vector abundance, resulting in different risks of an outbreak and of persistence in each of these areas, which can be visualised by risk maps. We created the maps to show the risk in different areas in the Netherlands of initial spread and of persistence. Furthermore, we investigated which vector-species contributes most to the spread by also creating individual risk maps for *Cx. pipiens* and *Ae. vexans*.

Risk maps are created by overlaying input data combined by specific calculations. Vector abundance (Figure 2) is based on the geographic and climatological features of a grid cell [19]. The host abundance for each grid cell are the cattle, sheep and goats located in that grid cell based on data of the Ministry of Economic Affairs, Agriculture & Innovation (Figure 2). The host and vector abundance and the estimates of the other parameter values are input for the model. The model includes aspects of vector biology, ecology, and virology.

Initial spread of RVFV is assessed at three moments during the vector season: 30 days after the start of the vector season (21^st^ May), half way (23^rd^ July), and 30 days before the end of the vector season (23^rd^ September). Persistence of the infection is determined by the Floquet ratio, *R*_*T*_, which summarizes growth over multiple years.

Rather than presenting a binary map with areas above or below the threshold, we depict maps with the probability that the initial growth rate *r* or the Floquet ratio, *R*_*T*_, exceeds the threshold. This probability is based on the accuracy and certainty of the estimated parameters within biological plausible ranges (see Additional file 1).

### Uncertainty analysis

In the uncertainty analysis, the model outcomes are calculated with different parameter values sampled by Latin Hypercube sampling [33] from their biological plausible interval. The range in outcomes reflects the magnitude of uncertainty introduced in the model outcome by the uncertainty in parameter estimates.

Twenty-five parameters were analysed in the uncertainty analysis. The 21 basic parameters of the RVF model, the ratio between *Cx. pipiens* and *Ae. Vexans,* the ratio between *Cx. pipiens s.l.* and *Cx. pipiens molestus,* the percentage of refractory hosts and the vector-host ratio. The vector-host ratio is varied in the uncertainty analysis by sampling from the vector-host ratios observed in the grids of 5 × 5 km areas. Additionally, the uncertainty in the estimation of mosquito abundance is taken into account by changing the estimated value by 10 fold (smaller or larger) using an extra parameter, with which the original vector-host ratio was thus multiplied.

The correlation between outcome of the model and each of the sampled parameter values is determined by the Kendall rank correlation coefficient (KRC) [34,35]. KRC coefficients of −1 or 1 represent a perfect correlation of outcome with parameter, correlation of 0 means no correlation.

## Results

### Risk maps

**Figure 3:**
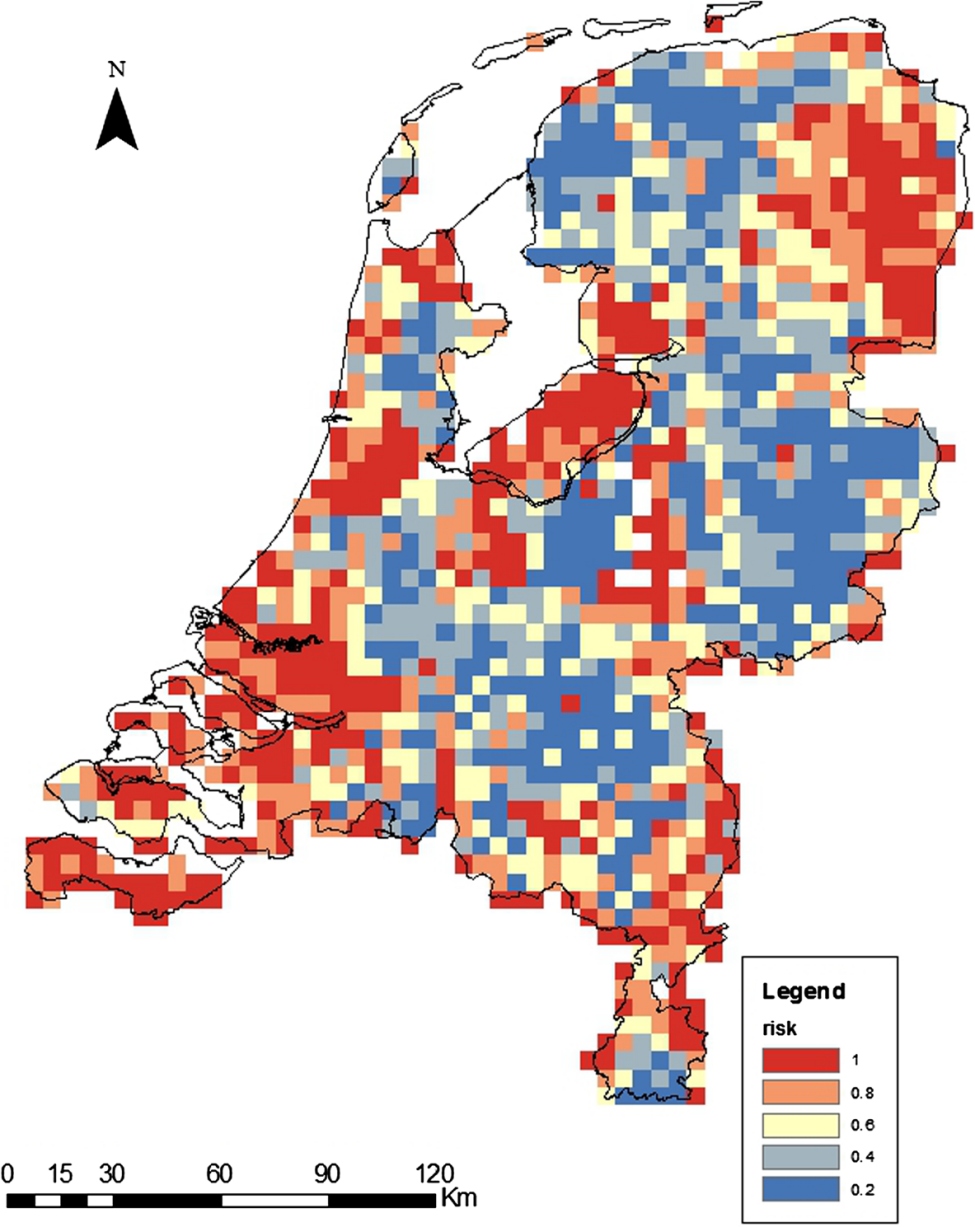
**Risk of persistence of RVFV in the Netherlands.** Blue indicates a low probability (< 20%) of the Floquet ratio *R*_*T*_ exceeding the threshold of 1, and red indicates a high probability (> 80%).

The map for risk of persistence of RVFV in the Netherlands (Figure 3) shows that the areas with high host abundance (Figure 2) have the lowest risk of a persistent RVFV infection, due to the low vector-host ratio.

**Figure 4:**
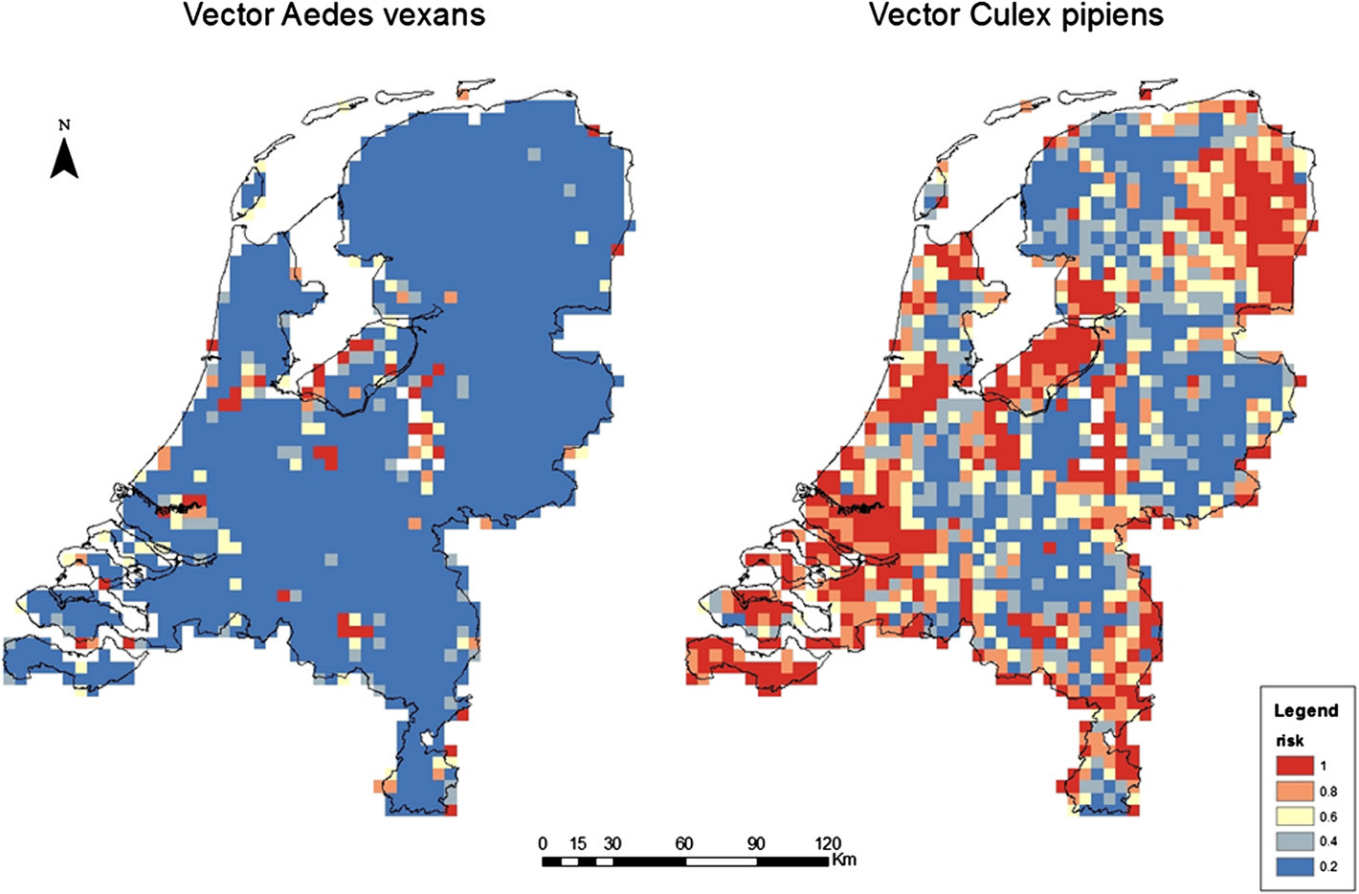
**Risk of persistence of RVFV in the Netherlands if the indicated vector is the only competent RVFV vector in the country.** Blue indicates a low probability (< 20%) of the Floquet ratio *R*_*T*_ exceeding the threshold of 1, and red indicates a high probability (> 80%).

The risk of persistence of RVFV is almost completely due to the presence of *Cx. pipiens* as shown by the map with *Cx. pipiens* being the only vector (Figure 4). The low abundance of *Ae. vexans* (Figure 2) in the Netherlands results in a minimal risk posed by this vector (Figure 4).

**Figure 5:**
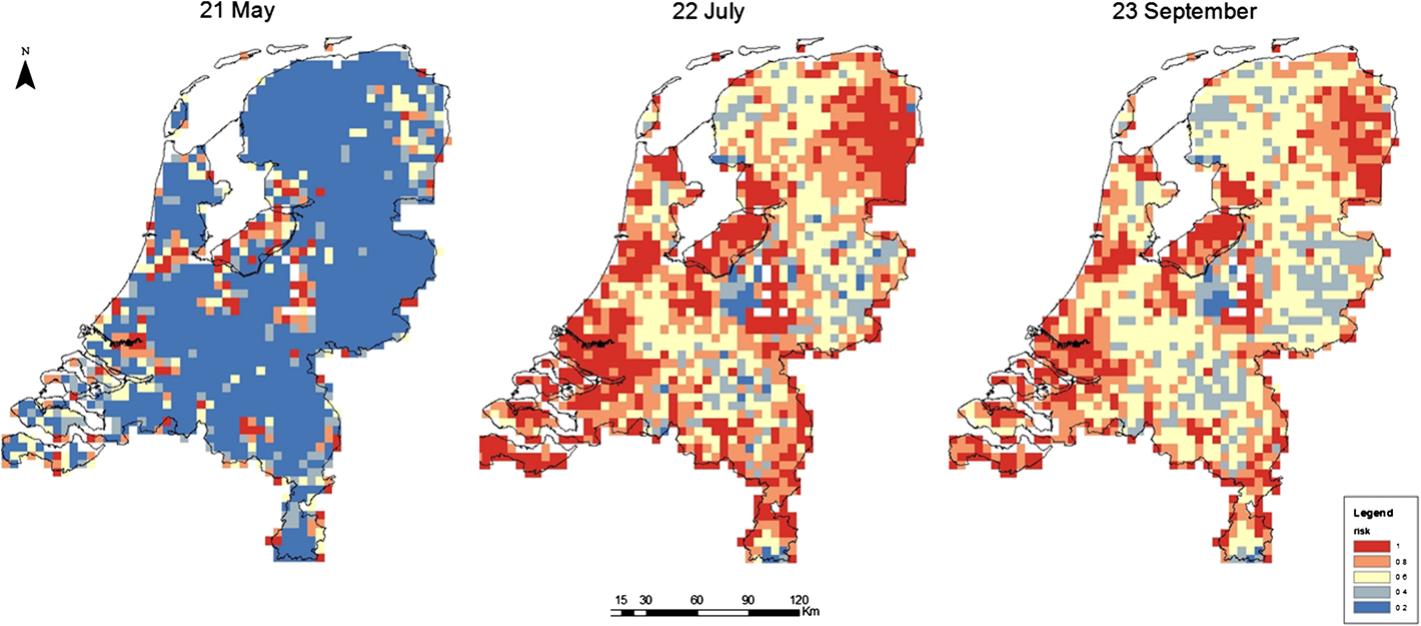
**Risk of RVFV outbreaks in May, July and September.** Blue indicates a low probability (< 20%) of the exponent of the initial epidemic growth rate (*e*^*r*^) exceeding the threshold of 1, and red indicates a high probability (> 80%). Day 1 (21 April) is the beginning of the season in which mosquitos are active.

The risk of outbreaks is higher halfway (July) and around the end (September) of the vector season than at the beginning (Figure 5). This is caused by higher vector abundances (see Additional file 1) and by differences in temperature affecting some model parameters of the vector. Autumn is a risk period for outbreaks as well, but these outbreaks will be cut short by the decline in mosquito abundances later in that year.

### Uncertainty analysis

**Figure 6:**
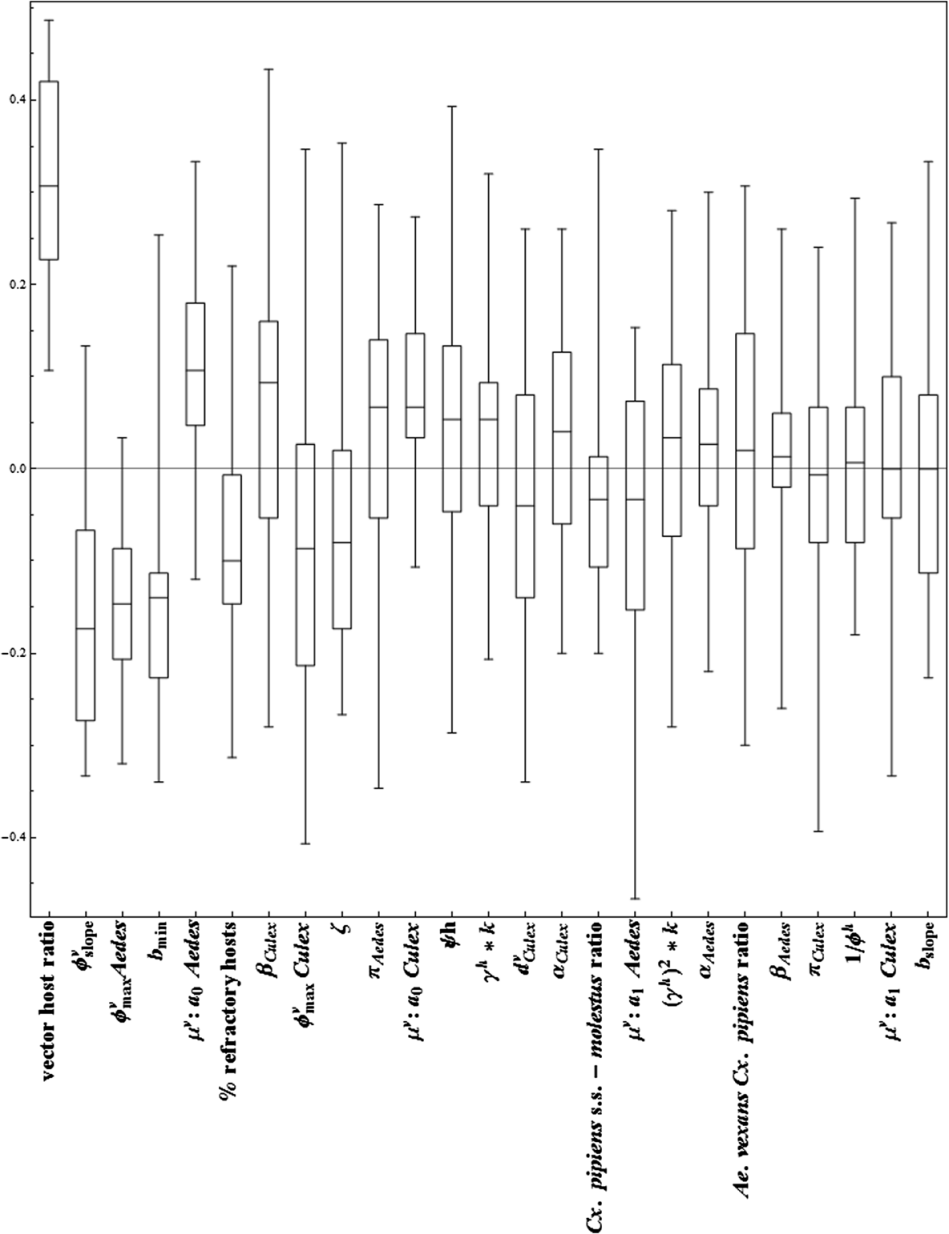
**Kendall Rank Correlation coefficients for 23 parameters in the uncertainty analysis.** Parameters are orderd by their absolute KRC value. For the explanation of parameters, see Table 1.

The vector-host ratio has the strongest effect (Figure 6) due to the high level of uncertainty in vector abundance (caused by the step from observed trap catch to the estimated number per km^2^, see Additional file 1). Also parameters associated with the probability of virus transmission by the vector (i.e. extrinsic incubation period (EIP), the biting rate and vector mortality) are important.

**Figure 7:**
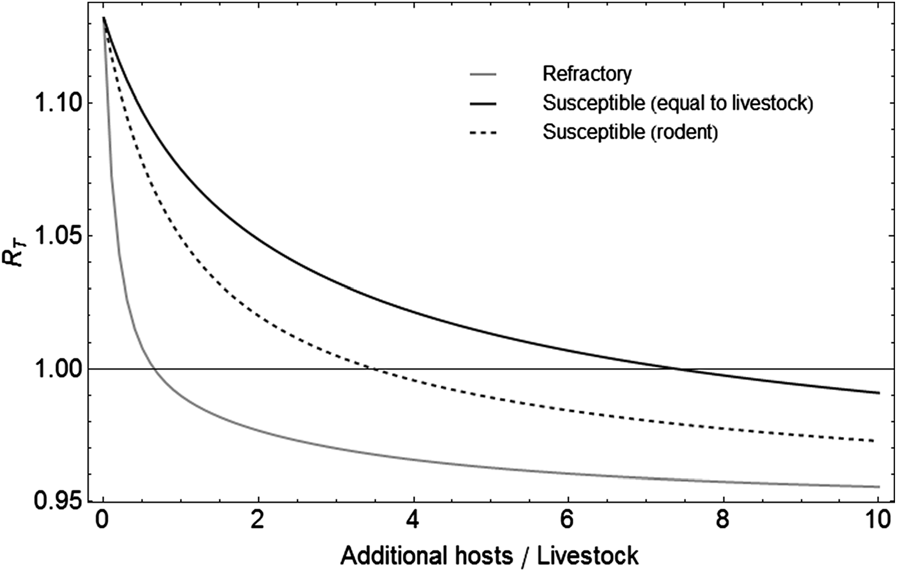
**Effect of non-livestock hosts on the Floquet ratio *R***_***T ***_**for a sparsely populated livestock area.** The *R*_*T*_ was calculated for an area with livestock density of 200 animals per 5 × 5 km and on the x-axis the number of additional hosts per livestock host. Additional hosts were either refractory (i.e. birds), susceptible with shorter infectious period (i.e. rodents), or susceptible and equal to livestock characteristics.

When the vectors are biting on animals other than livestock, the *R*_*T*_ is lower than without additional hosts. If these animals are refractory, i.e. resistant to infection, the *R*_*T*_ is lower than when these animals also contribute to the transmission of RVFV. For our sparsely populated livestock area (Table 2) the *R*_*T*_ crosses the threshold when the number of refractory hosts is 0.65 times the number of livestock animals, while this occurs if the number of rodent hosts exceeds 3.48 times the number of livestock animals. If the number of livestock (or animals which have epidemiological equal properties) increases by 7.39 times the threshold is crossed as well (Figure 7).

**Table 2:** **Characteristics (in number per 5 by 5 km area) of a sparsely (SPLA) and a densely (DPLA) populated livestock area in the Netherlands, and the resulting Floquet ratio *R***_***T***_

**Area**	**Cattle**	**Sheep & goat**	**Ae. vexans**	**Cx. pipiens**	**Vector-host ratio**	**Floquet ratio *R***_***T***_
SPLA	125	75	3100	32500	178	45 10^12^
DPLA	11625	2875	3100	32500	2.5	6 10^-4^

Mosquito densities are obtained from the modelled data and areas are selected with equal numbers of vectors.

## Discussion

The mathematical model developed in this paper provides insight into the spatial risk (risk maps) and major uncertainties of the potential risk of a Rift Valley fever outbreak in the Netherlands.

Counter-intuitively, the areas at risk of RVFV are predominantly the sparsely populated livestock areas (SPLAs, Figure 6) in which vector-host ratios are high. No other sources than cattle, sheep and goats are assumed in the area, such as wildlife or birds, to be present for a blood meal of the mosquitoes, thus leading to these high vector-host ratios. This results in a high probability of an outbreak occurring, because an initial infected host is bitten by many vectors. Stochastic fade-out or depletion of susceptible hosts is in SPLAs very likely to occur within the vector-season. Spill-over to densely populated livestock areas (DPLAs) contributes to an increased national impact of an outbreak in an SPLA. However, spatial spread between areas was not taken into account here.

The negative relationship between livestock density and the probability of an outbreak occurring is well known from other mathematical models of vector-borne infections (e.g. [36]). Also, within the definition of the vector-capacity, i.e. the potential of a vector-population to transmit a disease, includes the vector-host ratio [37]. This high sensitivity to the vector-host ratio was not observed in previous model studies of the potential of a major RVFV outbreak, because these studies did not include this parameter in their analyses [10,17]. However, our results do confirm the importance of the vector-lifespan found in these studies. The sensitivity to transmission probabilities found by Gaff et al. [10] was not observed in our analyses, and might have been obscured by the high influence of the vector-host ratio. In the bluetongue epidemic in France it has also been observed that an increase in cattle density resulted in lower seropositivity [38], indicating that higher host densities decrease the transmission potential. Due to the absence of outbreaks in areas similar to the Netherlands it is impossible to directly determine the risk of an outbreak, therefore this risk needs to be determined from underlying mechanisms. Mathematical models are a good tool to systematically follow an inductive line of reasoning [39]. In our model this meant that we needed to translate information from Africa to the situation in the Netherlands. Mortality rates of animals are likely to be much higher in naïve European herds than in African herds [4]. However, the uncertainty analyses showed that this had little impact on our results. Also, other factors that might be influenced by difference in susceptibility between breeds, i.e. transmission probability and length of the infectious period, were not found to have a substantial impact on the results.

The model was applied to the Netherlands, but this country can represent other areas in the temperate zone of at least northwest Europe. Livestock densities in some areas of the Netherlands are very high, but in other areas they are low (Figure 2). Our results indicate that areas with similar livestock and environmental characteristics should be considered at risk of RVFV outbreaks. Application of the model to other areas is possible when population dynamics of vectors and vector abundances are known.

The overwintering strategies of viruses, such as RVFV, in vectors cannot be determined in temperate zone, as long as no outbreaks have occurred. For instance, the bluetongue virus serotype 8 (BTV-8) overwintered in the Netherlands, Belgium and Germany unexpectedly, and it is still not clear how. For BTV-8 we do know that little happens in winter, and very quickly after reappearance of the vectors, the epidemic reappeared. *Cx. pipiens* s.l. is the main driver of a RVF-outbreak and can overwinter in the adult stage [40] and thus, infectious vectors can immediately become active again during the favourable season. Obviously there is a gradual decline in vector activity with declining temperature, and a gradual increase with increasing temperature. We modelled this in the form of a very abrupt end of transmission and an abrupt reappearance (stasis during the vector-free season), which, given the right choice of this winter period, does hardly affect the results (see Additional file 1: Figure S4). If RVFV cannot overwinter, persistence in the temperate zone is not possible, and the calculations of persistence (*R*_*T*_) are irrelevant. Based on the overwintering of BTV in Europe and the survival of RVFV during inter-epidemic periods in East-Africa, however, assuming survival during the winters is not unlikely.

Direct transmission of RVFV between mammals has been suggested [41], but never been experimentally proven to exist. Direct transmission increases the transmission rate locally (see Additional file 1: Figure S3), which could cause survival of the virus in absence of vectors (e.g. during the winter). Furthermore, direct transmission increases the outbreak potential such that densely populated livestock areas might be at risk of an outbreak.

*Cx. pipiens* contributes by far most to the spread and persistence of RVFV in the Netherlands (Figure 4), because it is by far the most common mosquito in the country. As a result, the uncertainty analysis of the model output shows that the results are strongly correlated with the estimated abundance of this vector population.

Here, *Cx. pipiens* is modelled as purely biting on mammals (livestock), and not on birds. The vector capacity for RVFV among livestock decreases if this mosquito takes blood meals from birds as well, which are not hosts for RVFV. Part of the population of *Cx. pipiens* might be ornithophilic (being *Cx. pipiens* s.s.) and the exact composition of the mosquito species complex in The Netherlands is still unknown (pers. com. E.J. Scholte). Populations from the south of France are known to be competent [14], but vector competence of the *Cx. pipiens* s.l. population in the Netherlands is unknown. Also when other animals, such as wildlife or rodents, are present in sparsely populated livestock areas, this may decrease the transmission potential substantially. Less than one refractory host per livestock animal (i.e. 0.65) is required to reduce the spread potential to values below the threshold. If refractory hosts are birds, it is likely that the value of 0.65 additional refractory host per livestock animal is exceeded and the threshold for spread is not reached. This will only occur, when vectors will be actually biting these refractory hosts. The model and thus the risk maps are in such a case an overestimation of the risk of a Rift Valley fever outbreak. Investigation into the vector properties of the major potential vector species for RVFV, *Cx. pipiens* s.l., is thus warranted.

Vertical transmission of the virus from adult *Ae. vexans* to eggs has almost no effect on an outbreak and even not on persistence of the infection in the Netherlands, because of the minor role of this vector species in the transmission (Figure 4). The existence and role of vertical transmission is subject to controversy. To our knowledge only one report [9] suggests the possibility of RVFV transmission via eggs in a related vector species, *Ae. linneatopennis*, which is not present in Europe. Studies that reproduce these findings under laboratory conditions are unknown [4].

Also unknown is the impact of the stable fly *Stomoxys calcitrans* (L.) which is highly efficient in transmitting the infection mechanically between hamsters in the laboratory [11]. In contrast to mosquitoes, stable flies are less likely to disperse over large areas, as their preferred breeding sites consist of straw, hay and manure [42]. Hence, stable flies do not have to leave a farm to find suitable breeding sites. The stable fly might act as an amplifying vector on a local farm if transmission between livestock hosts is as efficient as between hamsters: after introduction of a RVFV infection by a mosquito vector, the infection spreads very fast from animal to animal due to the presence of stable flies. Stable flies should be monitored during a RVFV epidemic to obtain valuable epidemiological data. However, whether these infected flies can be found during an entomological surveillance depends on the time between collection of vectors and virus detection, due to denaturation of the virus. The half life time of the virus in aerosols is only 6 h [43].

Another unknown actor is deer. Several deer species occur throughout Europe [44] and might play a role in the epidemiology of RVFV. Livestock in unaffected areas can get infected by migrating deer if these deer are infected. However, to our knowledge no deer species have been tested for RVFV competence and the preference of vectors for deer is unknown.

In summary, areas with a high vector to host ratio are most likely to experience an outbreak and persistence of the infection. The high vector-host ratio in the Netherlands is almost entirely due to the wide spread abundance of *Cx. pipiens* s.l. Our investigation underscores the importance to determine the vector competence and host preference of this mosquito species and others associated with cattle, sheep and goat.

## Abbreviations

Ae: *Aedes*; BTV(−8): Bluetongue virus (serotype 8); Cx: *Culex*; DPLA: Densely Populated Livestock Area; EIP: Extrinsic Incubation Period; EL&I: (Dutch) ministery of economic affairs, Agriculture and Innovation; I: Infectious class; KRC: Kendal Rank Correlation; L: Latently infected class; R: Recovered class; R0: Basic reproduction number; RT: Floquet ratio; RVF(V): Rift Valley fever (Virus); S: Susceptible class; s.l: Sensu lato; SPLA: Sparsely Populated Livestock Area; s.s: Sensu stricto.

## Competing interests

The authors declare that they have no competing interests.

## Authors’ contributions

EF has developed, programmed the computer code and analysed the model and drafted the manuscript. GB has collaborated in the development and interpretation of the model and developed the algorithms to calculate *R*_*T*_. He also produced the maps and was involved in drafting the manuscript. GN, AdK and HvR contributed to the development, parameterization of the model and interpretation of the results. They have critically revised the manuscript. All authors have read and approved of the final manuscript.

## Authors’ information

EF is a theoretical biologist with a PhD in Health Sciences. GB has a PhD in Biophysics and his work focusses on spatial epidemiology of veterinary infections. AdK is a theoretical biologist with a PhD in applied mathematics and is working on veterinary infectious disease modelling. GN is a DVM with a PhD in Veterinary Epidemiology and her work focusses on control of animal diseases and veterinary epidemiology. HvR has a PhD in modelling of insect population dynamics and is involved since then in veterinary epidemiology. All authors are researchers at the Central Veterinary Institute, part of Wageningen UR, in the Netherlands and are involved in both commercial, academic and government policy supporting research.

## Supplementary Material

Additional file 1 **Model descriptions, parameterization and analyses.** This additional file has five parts: describing the model, the parameterization, the reference curve used in the risk maps, and a sensitivity analyses on the assumption of direct transmission and of stasis during winter [4,7,9,11,15,19-21,25,27],[36,40,45-67]. Click here for file

## References

[B1] Daubney R Hudson JR Garnham PC Enzootic hepatitis or Rift Valley fever. An undescribed virus disease of sheep cattle and man from east Africa J Pathol Bacteriol 1931 34 545 579 10.1002/path.1700340418

[B2] Chevalier V Pepin M Plee L Lancelot R Rift Valley fever–a threat for Europe? Euro Surveill 2010 15 19506 20403309

[B3] Shoemaker T Boulianne C Vincent MJ Pezzanite L Al-Qahtani MM Al-Mazrou Y Khan AS Rollin PE Swanepoel R Ksiazek TG Nichol ST Genetic analysis of viruses associated with emergence of Rift Valley fever in Saudi Arabia and Yemen, 2000–01 Emerg Infect Dis 2002 8 1415 1420 10.3201/eid0812.020195 12498657 PMC2738516

[B4] Swanepoel R Coetzer JAW Coetzer JAW, Tustin RC Rift Valley fever Infectious diseases of livestock. Volume 2, edn 2004 Southern Africa: Cape Town: Oxford University Press

[B5] Davies FG Karstad L Experimental infection of the African buffalo with the virus of Rift Valley fever Trop Anim Health Prod 1981 13 185 188 10.1007/BF02237921 7344184

[B6] Evans A Gakuya F Paweska JT Rostal M Akoolo L Van Vuren PJ Manyibe T Macharia JM Ksiazek TG Feikin DR Breiman RF Kariuki Njenga M Prevalence of antibodies against Rift Valley fever virus in Kenyan wildlife Epidemiol Infect 2008 136 1261 1269 17988425 10.1017/S0950268807009806 PMC2870911

[B7] Easterday BC Murphy LC Bennett DG Experimental Rift Valley fever in calves, goats, and pigs Am J Vet Res 1962 23 1224 1230 13888969

[B8] EFSA Panel on Animal Health and Welfare Opinion of the scientific panel on animal health welfare on a request from the commission related to “the risk of a Rift Valley fever incursion and its persistence in the community” The EFSA Journal. EFSA 2005 238 1 128

[B9] Linthicum KJ Davies FG Kairo A Bailey CL Rift Valley fever virus (family bunyaviridae, genus phlebovirus). Isolations from Diptera collected during an inter-epizootic period in Kenya J Hyg (Lond) 1985 95 197 209 10.1017/S0022172400062434 2862206 PMC2129511

[B10] Gaff HD Hartley DM Leahy NP An epidemiological model of Rift Valley fever Electron J Diff Equ 2007 2007 1 12

[B11] Hoch AL Gargan TP 2nd Bailey CL Mechanical transmission of Rift Valley fever virus by hematophagous diptera Am J Trop Med Hyg 1985 34 188 193 3970308 10.4269/ajtmh.1985.34.188

[B12] Pepin M Bouloy M Bird BH Kemp A Paweska J Rift Valley fever virus (bunyaviridae: phlebovirus): an update on pathogenesis, molecular epidemiology, vectors, diagnostics and prevention Vet Res 2010 41 61 10.1051/vetres/2010033 21188836 PMC2896810

[B13] Gargan TP Clark GG Dohm DJ Turell MJ Bailey CL Vector potential of selected North American mosquito species for Rift Valley fever virus Am J Trop Med Hyg 1988 38 440 446 2895591 10.4269/ajtmh.1988.38.440

[B14] Moutailler S Krida G Schaffner F Vazeille M Failloux AB Potential vectors of Rift Valley fever virus in the Mediterranean region Vector Borne Zoonotic Dis 2008 8 749 754 10.1089/vbz.2008.0009 18620510

[B15] Turell MJ Linthicum KJ Patrican LA Davies FG Kairo A Bailey CL Vector competence of selected African mosquito (Diptera: Culicidae) species for Rift Valley fever virus J Med Entomol 2008 45 102 108 10.1603/0022-2585(2008)45[102:VCOSAM]2.0.CO;2 18283949

[B16] Rich KM Wanyoike F An assessment of the regional and national socio-economic impacts of the 2007 Rift Valley fever outbreak in Kenya Am J Trop Med Hyg 2010 83 52 57 10.4269/ajtmh.2010.09-0291 20682906 PMC2913501

[B17] Xue L Scott HM Cohnstaedt LW Scoglio C A network-based meta-population approach to model Rift Valley fever epidemics J Theor Biol 2012 306 129 144 22564391 10.1016/j.jtbi.2012.04.029

[B18] Mpeshe SC Haario H Tchuenche JM A mathematical model of Rift Valley fever with human host Acta Biotheor 2011 59 231 250 10.1007/s10441-011-9132-2 21611886

[B19] Ducheyne E Hendrickx G Abundance modeling of mosquito and biting midge species in the Netherlands 2010 Zoersel, Belgium: AVIA GIS 28

[B20] Royal Netherlands Meteorological Institute (KNMI) [http://www.knmi.nl/climatology/daily_data/download.html]

[B21] Madder DJ Surgeoner GA Helson BV Number of generations, egg-production, and developmental time of Culex pipiens and Culex restuans (Diptera, Culicidae) in southern Ontario J Med Entomol 1983 20 275 287 6876091 10.1093/jmedent/20.3.275

[B22] Sperlova A Zendulkova D Bluetongue: a review Vet Med-Czech 2011 56 430 452

[B23] Olive MM Goodman SM Reynes JM The role of wild mammals in the maintenance of Rift Valley fever virus J Wildl Dis 2012 48 241 266 22493102 10.7589/0090-3558-48.2.241

[B24] Diallo M Nabeth P Ba K Sall AA Ba Y Mondo M Girault L Abdalahi MO Mathiot C Mosquito vectors of the 1998-1999 outbreak of Rift Valley Fever and other arboviruses (Bagaza, Sanar, Wesselsbron and West Nile) in Mauritania and Senegal Med Vet Entomol 2005 19 119 126 10.1111/j.0269-283X.2005.00564.x 15958020

[B25] Jupp PG Kemp A Grobbelaar A Leman P Burt FJ Alahmedt AM AL Mujalli D AL Khamees M Swanepoel R The 2000 epidemic of Rift Valley fever in Saudi Arabia: mosquito vector studies Med Vet Entomol 2002 16 245 252 10.1046/j.1365-2915.2002.00371.x 12243225

[B26] Mondet B Diaite A Ndione JA Fall AG Chevalier V Lancelot R Ndiaye M Poncon N Rainfall patterns and population dynamics of Aedes (Aedimorphus) vexans arabiensis, Patton 1905 (Diptera: Culicidae), a potential vector of Rift Valley fever virus in Senegal J Vector Ecol 2005 30 102 106 16007962

[B27] Faran ME Turell MJ Romoser WS Routier RG Gibbs PH Cannon TL Bailey CL Reduced survival of adult Culex-pipiens infected with Rift Valley fever virus Am J Trop Med Hyg 1987 37 403 409 3661832 10.4269/ajtmh.1987.37.403

[B28] Bacaer N Gomes MG On the final size of epidemics with seasonality Bull Math Biol 2009 71 1954 1966 10.1007/s11538-009-9433-7 19475453

[B29] Boender GJ De Koeijer AA Fischer EAJ Derivation of a Floquet formalism within a natural framework Acta Biotheor 2012 60 303 317 10.1007/s10441-012-9162-4 22743961 PMC3440566

[B30] Edelstein-Keshet L Mathematical models in biology 1988 New York: Random House

[B31] Heesterbeek JAP Roberts MG Threshold quantities for infectious diseases in periodic environments J Biol Syst 1995 3 779 787 10.1142/S021833909500071X

[B32] Klausmeier CA Floquet theory: a useful tool for understanding nonequilibrium dynamics Theor Ecol 2008 1 153 161 10.1007/s12080-008-0016-2

[B33] McKay MD Beckman RJ Conover WJ A comparison of three methods for selecting values of input variables in the analysis of output from a computer code Technometrics 1979 21 239 245

[B34] Backer JA Nodelijk G Transmission and control of African horse sichness in The Netherlands: a model analysis PLoS One 2011 6 e23066 10.1371/journal.pone.0023066 21850252 PMC3151287

[B35] Kendall M A new measure of rank correlation Biometrika 1938 30 81 89

[B36] Hartemink N Purse BV Meiswinkel R Brown HE De Koeijer AA Elbers ARW Boender GJ Rogers DJ Heesterbeek JAP Mapping the basic reproduction number (R0) for vector-borne diseases: a case study of bluetongue virus Epidemics 2009 1 153 161 10.1016/j.epidem.2009.05.004 21352762

[B37] Garrett-Jones C Shidrawi GR Malaria vectorial capacity of a population of Anopheles gambiae: an exercise in epidemiological entomology Bull World Health Organ 1969 40 531 545 5306719 PMC2556109

[B38] Durand B Zanella G Biteau-Coroller F Locatelli C Baurier F Simon C Le Dréan E Delaval J Prengere E Beauté V Guis H Anatomy of bluetongue virus serotype 8 epizootic wave, France, 2007–2008 Emerg Infect Dis 2010 16 1861 1868 10.3201/eid1612.100412 21122214 PMC3294545

[B39] McKenzie FE Why model malaria? Parasitol Today 2000 16 511 516 10.1016/S0169-4758(00)01789-0 11121847

[B40] Bailey CL Faran ME Gargan TP 2nd Hayes DE Winter survival of blood-fed and nonblood-fed Culex-pipiens L Am J Trop Med Hyg 1982 31 1054 1061 6289686 10.4269/ajtmh.1982.31.1054

[B41] Chevalier V Rakotondrafara T Jourdan M Heraud JM Andriamanivo HR Durand B Ravaomanana J Rollin PE Rakotondravao R An unexpected recurrent transmission of Rift Valley fever virus in cattle in a temperate and mountainous area of Madagascar Plos Negl Trop D 2011 5 e1423 10.1371/journal.pntd.0001423 PMC3243698 22206026

[B42] Bishopp FC The stable fly (stomoxys calcitrans L.), an important live stock pest J Econ Entomol 1913 6 112 128

[B43] Miller WS Artenstein MS Aerosol stability of three acute respiratory disease viruses Proc Soc Exp Biol Med 1967 125 222 227 10.3181/00379727-125-32054 4290945

[B44] Apollonio M, Andersen R, Putman R European Ungulates and their Management in the 21st Century 2010 Cambridge: Cambridge University Press

[B45] Keeling MJ Rohani P Modeling infectious diseases in humans and animals 2008 Princeton, New Jersey, USA: Princeton University Press

[B46] McIntosh BM Dickinson DB Dos Santos I Rift Valley fever. 3. Viraemia in cattle and sheep. 4. The susceptibility of mice and hamsters in relation to transmission of virus by mosquitoes J S Afr Vet Assoc 1973 44 167 169 4149110

[B47] Olaleye OD Tomori O Schmitz H Rift Valley fever in Nigeria infections in domestic animals Rev Sci Tech 1996 15 937 946 9025143 10.20506/rst.15.3.966

[B48] Swanepoel R Struthers JK Erasmus MJ Shepherd SP Mcgillivray GM Shepherd AJ Hummitzsch DE Erasmus BJ Barnard BJH Comparative pathogenicity and antigenic cross-reactivity of Rift Valley fever and other African phleboviruses in sheep J Hyg (Lond) 1986 97 331 346 10.1017/S0022172400065426 3537119 PMC2083542

[B49] Carron A Bichaud L Platz N Bicout DJ Survivorship characteristics of the mosquito Aedes caspius adults from southern France under laboratory conditions Med Vet Entomol 2008 22 70 73 10.1111/j.1365-2915.2008.00718.x 18380656

[B50] Costello RA Brust RA Longevity of Aedes-vexans diptera-culicidae under different temperatures and relative humidities in laboratory J Econ Entomol 1971 64 324 325

[B51] Wonham MJ De-Camino-Beck T Lewis MA An epidemiological model for West Nile virus: invasion analysis and control applications Proc Biol Sci 2004 271 501 507 10.1098/rspb.2003.2608 15129960 PMC1691622

[B52] Gad AM Feinsod FM Soliman BA el Said S Survival estimates for adult Culex-pipiens in the Nile Delta Acta Trop 1989 46 173 179 10.1016/0001-706X(89)90034-X 2566270

[B53] Ba Y Diallo D Dia I Diallo M [Feeding pattern of Rift Valley fever virus vectors in Senegal. Implications in the disease epidemiology] Bull Soc Pathol Exot 2006 99 283 289 (in French) 17111980

[B54] Briegel H Waltert A Kuhn AR Reproductive physiology of Aedes (Aedimorphus) vexans (Diptera: Culicidae) in relation to flight potential J Med Entomol 2001 38 557 565 10.1603/0022-2585-38.4.557 11476336

[B55] Ndiaye PI Bicout DJ Mondet B Sabatier P Rainfall triggered dynamics of Aedes mosquito aggressiveness J Theor Biol 2006 243 222 229 10.1016/j.jtbi.2006.06.005 16876201

[B56] Meegan JM Khalil GM Hoogstraal H Adham FK Experimental transmission and field isolation studies implicating Culex pipiens as a vector of Rift Valley fever virus in Egypt Am J Trop Med Hyg 1980 29 1405 1410 7446827 10.4269/ajtmh.1980.29.1405

[B57] Turell MJ Gargan TP 2nd Bailey CL Replication and dissemination of Rift Valley fever virus in Culex pipiens Am J Trop Med Hyg 1984 33 176 181 6696176 10.4269/ajtmh.1984.33.176

[B58] Turell MJ Gargan TP 2nd Bailey CL Culex pipiens (Diptera, Culicidae) morbidity and mortality associated with Rift Valley fever virus infection J Med Entomol 1985 22 332 337 4009630 10.1093/jmedent/22.3.332

[B59] Patrican LA Bailey CL Ingestion of immune bloodmeals and infection of Aedes fowleri, Aedes mcintoshi, and Culex pipiens with Rift Valley fever virus Am J Trop Med Hyg 1989 40 534 540 2729509 10.4269/ajtmh.1989.40.534

[B60] Turell MJ Presley SM Gad AM Cope SE Dohm DJ Morrill JC Arthur RR Vector competence of Egyptian mosquitoes for Rift Valley fever virus Am J Trop Med Hyg 1996 54 136 139 8619436 10.4269/ajtmh.1996.54.136

[B61] Turell MJ Effect of environmental-temperature on the vector competence of Aedes taeniorhynchus for Rift Valley fever and Venezuelan equine encephalitis viruses Am J Trop Med Hyg 1993 49 672 676 8279634 10.4269/ajtmh.1993.49.672

[B62] Gad AM Hassan MM Elsaid S Moussa MI Wood OL Rift Valley fever virus transmission by different Egyptian mosquito species Trans R Soc Trop Med Hyg 1987 81 694 698 10.1016/0035-9203(87)90460-3 2895516

[B63] Hamer GL Kitron UD Goldberg TL Brawn JD Loss SR Ruiz MO Hayes DB Walker ED Host selection by Culex pipiens mosquitoes and West Nile virus amplification Am J Trop Med Hyg 2009 80 268 278 19190226

[B64] Medlock JM Snow KR Leach S Potential transmission of West Nile virus in the British Isles: an ecological review of candidate mosquito bridge vectors Med Vet Entomol 2005 19 2 21 10.1111/j.0269-283X.2005.00547.x 15752172

[B65] Gad AM Farid HA Ramzy RRM Riad MB Presley SM Cope SE Hassan MM Hassan AN Host feeding of mosquitoes (Diptera: Culicidae) associated with the recurrence of Rift Valley fever in Egypt J Med Entomol 1999 36 709 714 10593070 10.1093/jmedent/36.6.709

[B66] Platonov AE Fedorova MV Karan LS Shopenskaya TA Platonova OV Zhuravlev VI Epidemiology of West Nile infection in Volgograd, Russia, in relation to climate change and mosquito (Diptera: Culicidae) bionomics Parasitol Res 2008 103 Suppl 1 S45 S53 19030885 10.1007/s00436-008-1050-0

[B67] Takken W Verhulst N Scholte EJ Jacobs F Jongema Y Distribution and dynamics of arthropod vectors of zoonotic disease in The Netherlands in relation to risk of disease transmission. Report of project TRC2005/2867 of the Ministry of Agriculture, Nature Conservation and Food Security 2007 Wageningen: Laboratory of entomology, Wageningen UR 55

