# Supplementary material for: The transmission potential of Rift Valley fever virus among livestock in the Netherlands: a modelling study
Source: Vet Res. 2013 Jul 22;44(1):58. doi: 10.1186/1297-9716-44-58 (PMC3733972; doi:10.1186/1297-9716-44-58)
Supplement: Additional file 1 — Model descriptions, parameterization and analyses. This additional file has five parts: describing the model, the parameterization, the reference curve used in the risk maps, and a sensitivity analyses on the assumption of direct transmission and of stasis during winter [4,7,9,11,15,19-21,25,27],[36,40,45-67]. [file 1297-9716-44-58-S1.docx]

**Additional file 1 Model descriptions, parameterization and analyses**

# Outline of additional file 1

This additional file has five parts describing the model, the parameterization, the reference curve used in the risk maps and a sensitivity analyses on the assumption of direct transmission and of stasis during winter.

The additional file will start with an elaborate description of the model including the equations. Some parts of this description are duplicated from the main text to make it easier to read through the description without having to refer back to the main text. The second part of the ESI contains detailed information on the model parameterization. This includes data on temperature dependence, literature overview used to estimate parameters, and calculation of the vector and host population sizes. In the third part we include the calculations used to determine the probability of the values of *r* or *R_T_* exceeding the threshold of 1 using a reference curve. . In the fourth part we show the effect of direct transmission between hosts on the value of the Floquet ratio, *R_T_*. Finally, in the fifth part we show that assuming stasis for host and vector does not affect the results much from assuming stasis in the vector alone. Therefore, we could use the more convenient assumption that both host and vector are in stasis during the vector-free winter season.

# Part 1: Model equations

## Ordinary differential equations

The core of the model is a set of coupled ordinary differential equations (ODEs, Equations 1-13). Equations 1-6 describe the dynamics of the infection in hosts of species *j*, in which superscript *h* indicates that the variable depicts a host and likewise, superscript *v* depicts a variable representing a vector. Host are categorized into four states: susceptible (*S^h^*), latent (*L^h^*), infectious (*I^h^*) and recovered (*R^h^*). The latent and infectious state is divided into sub-classes to allow for a gamma-distributed latent and infectious period [45].

| $\begin{matrix} \frac{dS_{j}^{h}}{dt}=-\sum_{i=1}^{m} \left(\Lambda_{ij}^{h}b_{i}(t)\frac{S_{j}^{h}}{N_{j}^{h}}I_{i}^{v}\right) \\ \frac{dL_{j}^{h}}{dt}=\sum_{i=1}^{m} \left(\Lambda_{ij}^{h}b_{i}(t)\frac{S_{j}^{h}}{N_{j}^{h}}I_{i}^{v}\right)-\varphi_{j}^{h}L_{j}^{h} \\ \frac{dI_{j}^{1}}{dt}=\varphi_{j}^{h}L_{j}^{h}-\gamma_{j}I_{j}^{1} \\ \frac{dI_{j}^{g}}{dt}=\gamma_{j}I_{j}^{g-1}-\gamma_{j}I_{j}^{g} \\ I_{j}^{h}=\sum_{i=0}^{k_{j}} I_{j}^{i} \\ \frac{dR_{j}^{h}}{dt}=\gamma_{j}I_{j}^{k} \end{matrix}$ | Equation 1 |
| --- | --- |
|  | Equation 2 |
|  | Equation 3 |
|  | Equation 4 |
|  | Equation 5 |
|  | Equation 6 |

We start the explanation with the simplest Equations 3-6 describing the *k* infectious classes (*I^h^*) and the recovered state (*R^h^*). Latently infected hosts (*L^h^*) enter the first infectious state in the first infectious class from the latent state with a transition rate $\varphi_{j}^{h}$, and leave each infectious class with transition rate $\gamma_{j}^{h}$to the next infectious class. The hosts in the last infectious class *k* enter into the recovered state. The hosts remain in this state until they die, and are replaced by birth of new susceptible animals.

More complicated is the calculation of the transmission rate which is the rate at which hosts are infected, thus transit from the susceptible (*S^h^* ) to the latent state (*L^h^*). This rate is given by the rather cryptic factor$\sum_{i=1}^{m} \left(\Lambda_{ij}^{h} b_{i}(t) (S_{j}^{h}/N_{j}^{h})\cdot I_{i}^{v}\right)$, which sums the infection pressure from all infected vectors of *m* vector species. This summation includes the number of infectious vectors *I^v^_j_* and the fraction of susceptible hosts $S_{j}^{h}/N_{j}^{h}$, the biting rate *b_i_(t)* of vector *i* and the term $\Lambda_{ij}^{h}$. The term $\Lambda_{ij}^{h}$ is the *host specific per bite transmission* from vector *i* to host *j*, which is defined as the fraction of successful transmission events from one infected vector of species *i* to a host of species *j* per bite.

$\begin{matrix} \Lambda_{ij}^{h}=\alpha_{ij}\cdot p_{ij} \\ \begin{matrix} & \end{matrix}=\alpha_{ij}\frac{\pi_{ij}N_{j}^{h}}{\sum_{k=1}^{n} \pi_{ik}N_{k}^{h}} \end{matrix}$ Equation 7

This term includes the transmission probability $\alpha_{ij}$from vector species *i* to host species *j*, and the probability *p_ij_* of biting a host of species *j*. The probability of biting a host of species *j* is calculated by multiplication of the preference $\pi_{ij}$for host *j* by vector species *i* with the number of hosts of species *j* (*N _j_*), divided by the sum of all preferences times host population sizes. We emphasize that the preferences *and* host population sizes determine the distribution of bites of a certain vector species over the host species. The biting rate (number of bites per vector per day) is not affected by the number of hosts nor is the composition of hosts of influence on the biting rate of an individual vector.

A distinction is made between two types of vectors: vectors with vertical transmission from adult to eggs (*Aedes vexans*), and vectors without vertical transmission (*Culex pipiens*).

We assume that uninfected vectors are all susceptible. Vectors have a latent state (in entomology called the extrinsic incubation period) in which they cannot infect hosts, and subsequently an infectious state in which they can infect hosts. The infected vectors remain infectious until death. A fraction of eggs of infectious vertical transmitting vectors will become infected. After hatching and passing through larval states, these eggs develop into infectious vectors. Infected eggs form an extra infected state *Y*. The dynamics of virus replicating vectors with vertical transmission are then described by equations 8-12. For virus replicating vector without vertical transmission, the equations are the same except that eggs do not become infected.

| $\begin{matrix} \frac{dN_{i}^{v}}{dt}=h_{i}(t)-\mu_{i}^{v}(t)N_{i}^{v} \\ \frac{dY_{i}}{dt}=\varsigma_{i}c_{i}b_{i}(t)I_{i}^{v}-h_{i}(t)\frac{Y_{i}}{G_{i}} \\ \frac{dS_{i}^{v}}{dt}=-\sum_{i=1}^{n} \left(\Lambda_{ij}^{v}b_{i}(t)S_{i}^{v}I_{j}^{h}\right)+h_{i}(t)\frac{G_{i}-Y_{i}}{G_{i}}-\mu_{i}^{v}(t)S_{i}^{v} \\ \frac{dL_{i}^{v}}{dt}=\sum_{j=1}^{n} \left(\Lambda_{ij}^{v}b_{i}(t)S_{i}^{v}I_{j}^{h}\right)-(\mu_{i}^{v}(t)+\varphi_{i}^{v}(t))L_{i}^{v} \\ \frac{dI_{j}^{v}}{dt}=h_{i}(t)\frac{Y_{i}}{G_{i}}+\varphi_{i}^{v}(t)L_{i}^{v}-(\mu_{i}^{v}(t)\cdot d_{i}^{v})I_{i}^{v} \end{matrix}$ | Equation 8 |
| --- | --- |
|  | Equation 9 |
|  | Equation 10 |
|  | Equation 11 |
|  | Equation 12 |

The first equation gives the vector population dynamics. *N^v^_j_* is the population size of vector *i*. The vector population size depends on the number of new adult vectors entering the population *h_i_(t)* and the mortality of vectors *µ^v^_i_(t)*.

The total number of eggs present in the environment is difficult to observe in the field and no data for the Netherlands is available to us. To incorporate infected eggs in the model we made some simplifying assumptions: (1) we only model eggs that will eventually develop into an adult vector (so egg, larval and pupal mortality is not explicitly modelled), (2) the number of produced eggs equals the number of hatching adults during a year, such that the adult vector population size remains equal each year, and (3) the number of eggs is constant in time. Using these simplifications in egg and adult vector population dynamics, we modelled the impact of vertical transmission.

Vectors with vertical transmission produce infected eggs with a probability $\varsigma_{i}$. The rate at which one female produces eggs, is determined by the biting rate *b_i_(t)* and the batch size *c_i_*. The batch size *c_i_* is determined in the model such that the vector population remains equal for each year (see assumption 1 and 2). The eggs hatch with a number of *h_i_(t)* at time *t*. To determine the fraction of infected (hatching) eggs, we assume that the egg population is constant during the year at size *G_i_* (see assumption 3). The size of the egg population *G_i_* is estimated by the inverse of the mean egg survival time multiplied by the maximum adult vector population abundance, i.e. the peak abundance during the season. For non-vertical transmitting vectors, the eggs are disregarded in the model.

Different from the infectious state in hosts, for virus replicating vectors the infectious state is ended only by death of the vector. In some vector species the mortality rate increases with a factor *d_i_^v^* due to infection.

The transmission rate to vectors is determined by the summation of infection by different host species. This calculation includes the number of infectious hosts *I^h^_j_* and the number of susceptible vectors, the biting rate *b_i_(t)* of vector *i* and the factor $\Lambda_{ij}^{v}$. This factor $\Lambda_{ij}^{v}$ is the *per bite transmission* from one infected individual of host *j* to a susceptible individual of vector *i*, which is defined as the fraction of successful transmission events during one bite on a random host of species *j* by a random vector of species *i*:

| $\begin{matrix} \Lambda_{ij}^{v}=\beta_{ij}\cdot p_{ij}\cdot\frac{1}{N_{j}^{h}} \\ \begin{matrix} & \end{matrix}=\beta_{ij}\frac{\pi_{ij}}{\sum_{k=1}^{n} \pi_{ik}N_{k}^{h}} \end{matrix}$, | Equation 13 |
| --- | --- |

where $\beta_{ij}$ is the transmission probability from host to vector during one bite and *p_ij_* is the probability of biting a host of species *j* by vector species *i* . This probability *p_ij_* depends on the vector preference and host abundances (Equation 7). The probability of biting the *one* infectious host is given by dividing with the host population size: $1/N_{k}^{h}$.

## Transmission and removal matrices

To calculate the threshold criteria for the stability of the disease free equilibrium at a specific point in time and for the Floquet ratio, R_T_. The system of ordinary differential equations is rewritten into two matrices; the transmission matrix **T** and the removal matrix **D**. As an example the transmission matrix **T** and removal matrix **D** for our model with only one vector and one host and the simplification of only one infectious state for hosts will be shown:

$\frac{d}{dt} \left( \begin{matrix} \begin{matrix} \begin{matrix} Y \\ L^{v} \\ I^{v} \end{matrix} \\ L^{h} \\ I^{h} \end{matrix} \end{matrix} \right)=\mathbf{T}-\mathbf{D}$ **=**

$$\left( \begin{matrix} 0 & 0 & \zeta c b(t) & 0 & 0 \\ 0 & 0 & 0 & 0 & \Lambda^{v}b\left( t \right)S^{v} \\ \frac{h\left( t \right)}{G} & \phi^{v}(t) & 0 & 0 & 0 \\ 0 & 0 & \Lambda^{h}b\left( t \right)\frac{S^{h}}{N^{h}} & 0 & 0 \\ 0 & 0 & 0 & \phi^{h} & 0 \end{matrix} \right)-\left( \begin{matrix} \frac{h\left( t \right)}{G} & 0 & 0 & 0 & 0 \\ 0 & \mu^{v}(t)+\phi^{v}(t) & 0 & 0 & 0 \\ 0 & 0 & {d^{v} \mu}^{v}(t) & 0 & 0 \\ 0 & 0 & 0 & \phi^{h} & 0 \\ 0 & 0 & 0 & 0 & \gamma\end{matrix} \right)$$

# Part 2: Parameterization

## Average daily temperature

Several parameters related to the vector, such as the vector mortality, biting rate and extrinsic incubation period, depend on the temperature. We assumed that the average daily temperature (24 h average) is adequate to describe this temperature dependence. The average daily temperature for De Bilt in the Netherlands (, in °C) is obtained from the Royal Dutch Meteorological Institute KNMI (data of 1971-2000) [55].


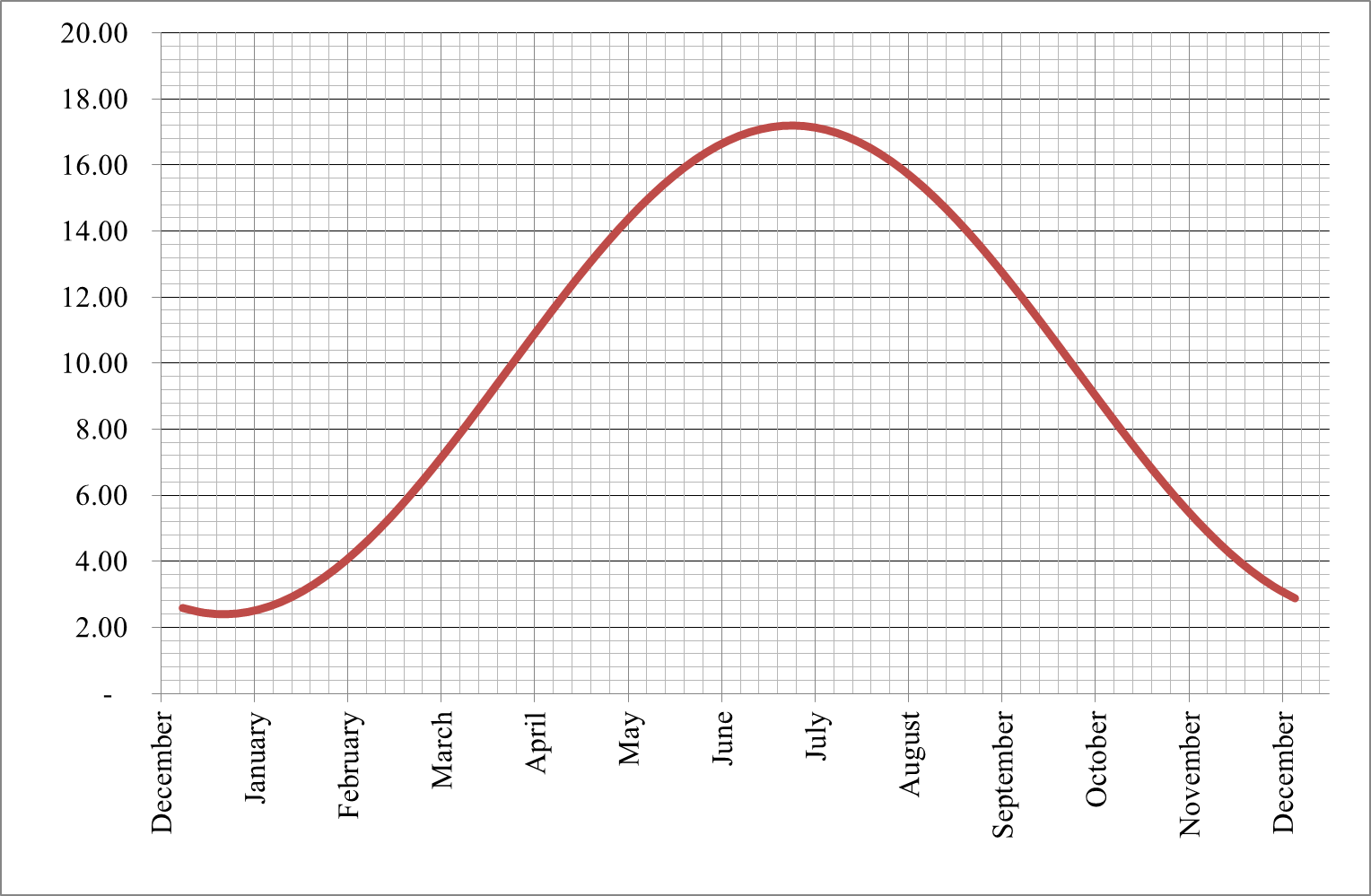


**Figure S1 Average daily (24 h) temperature in °C measured in De Bilt, the Netherlands in the period 1971-2000.**

## Host parameters

### Longevity

Cattle, sheep and goats are kept for several years on a farm. Because this is a much larger time scale than the duration of the infectious period of RVF, we assumed that these animals not to be removed or die, and these populations are thus constant.

### Infection parameters

Cattle become viraemic at 1-2 days post infection, and the viraemia peaks at 2-5 days post infection [4, 7]. The viraemia remains detectable up to 7 days, but for calves 5.9 days [46].

For Nigerian sheep breeds fever and viraemia was found after 24 h, which remained present up to 7 days. The sheep of one breed (Yankasa) all died during the viraemic period [47]. Lambs younger than one week at infection showed viraemia after 16 h and died between 36 and 42 h [4,7]. Older lambs were viraemic for up to 3 days, and at the next sample 7 days later they were negative [46]. In older sheep and goat, viraemia was found 1-2 days after inoculation, also peaking at 2-5 days. The virus was detectable up to 7 days [4,7,48].

For the calculations we considered the latent period and the infectious period of cattle, sheep and goat to be equal. Overall, the data imply that the latent period is 1 day and the infectious period is 5 days with a variance of 1.25 days ().

## Vector parameters

### Longevity

Survival studies indicate that, given a constant temperature, an exponential distribution of the longevity of mosquitoes (i.e. duration of adult stage) is a good description [40,49]. Therefore we assumed that the longevity can be described by one parameter *μ^v^* for each of the species, and the average longevity is 1/ *μ^v^*. This parameter does, however, change with temperature.

This average longevity of mosquitoes is negatively correlated with temperature, described by a linear decrease (see equation in ). The longevity of both *Aedes* *vexans* is based on data at constant temperatures of 13 °C and 21 °C [50]. The longevity of *Cx. pipiens* s.l. is over 30 days at temperatures below 20 °C [51], declining to 10 or 14 days at 24-27 °C [52].

Infection by RVFV increases the mortality rate of *Cx. pipiens* s.l. with 26% [27]. This is not the case for *Aedes* *vexans*.

### Biting rate

Mosquito females take a blood meal to develop eggs. Hence the time between two blood meals consists of the total time to mature eggs, to find a breeding site and to oviposit (lay eggs). This cycle is called the gonotrophic cycle. The largest proportion of the gonotrophic cycle consists of maturation of the eggs and the maturation of the eggs is temperature dependent. The length of the gonotrophic cycle for *Cx. pipiens* s.l. as function of temperature was estimated at laboratory and natural conditions [21]. For *Ae. vexans* several African and European estimates were made, but none report the temperature. However, the few available data points for *Ae. vexans* correspond to that for *Cx. pipiens* s.l., taking the long term daily average temperatures in the area of study [53-55]. Therefore, the biting rate for all mosquito species is taken equal.

Mosquito biting activity seizes at 9.6 °C (i.e. biting rate is zero) and the biting rate increases by 0.0173 day^-1^ T^-1^ [21].

### Extrinsic incubation period

The extrinsic incubation period (EIP), equivalent to the latent period in hosts, is the time between a blood meal on an infectious host and the first successful virus transmission from vector to host during another blood meal. The EIP depends on virus replication and external temperature. The length of the EIP was fitted to experimental data for *Cx. pipiens* s.l. [56-58] and *Ae. vexans arabiensis* [25]. The temperature dependence of the EIP for *Ae. vexans arabiensis* could not be estimated, as the two data points were at temperatures only a few degrees apart. The same slope (-0.30 day T^-1^) of linear relationship with temperature as for *Cx. pipiens* s.l. was used [56-58]. The maximum biting rate was 18.9 day^-1^ for *Aedes* [25] and 11.3 day^-1^ for *Culex* [56-58].

### Duration of the infectious period

The infectious period is ended by death for vectors that replicate the virus and is thus equal to the life expectancy at the moment of infection with RVFV.

## Host-vector interaction

Host-vector interactions consist of the parameters described in Equations 7 and 13, which are transmission probabilities from vector to host $\alpha_{qj}$ and from host to vector $\beta_{qj}$, and the host preference of a vector $\pi_{qj}$.The estimates of the transmission probabilities were based on laboratory studies with mosquitoes and RVFV infected and uninfected hamsters. Host preference was based most preferably on choice experiments with different host species, and if not available, on analysis of the vector’s gut content. However, from blood meal analysis the *preference* of the vector cannot be determined accurately, because the content of the gut is the result of a combination of host preference and host availability (i.e. host density).

### Transmission probabilities

The transmission probability from host to virus replicating vector is determined as the fraction of disseminated infected vectors after a blood meal. Virus isolation from the legs of arthropods (after disinfection of the outside) indicates that the infection has disseminated through the body of the vector.

#### Aedes vexans

The transmission probabilities from and to *Aedes* species were determined by experiments with *Ae. mcintoshi, Ae. fowleri, Ae. taeniorhynchus* and *O. caspius.* Unfortunately, *Ae. vexans arabiensis* mosquitoes were tested in a pool such that only competence and not transmission probability per bite could be calculated [25].

Transmission from host to *Aedes* species ranged between 18% and 82% having a disseminated infection. These values were derived from three studies. Infection and dissemination was 30% for *Ae. fowleri*, 60% for *Ae. mcintoshi* [59] and 40% for *O. caspius* [60]. It was shown that rearing temperature had an effect for *Ae. taeniorhynchus,* with dissemination rates ranging from 18% and 60% [61]. *O. caspius* was infected (not clear whether disseminated or not) in 77.5% to 82.14% of the cases after one blood meal [62].

Transmission from *Aedes* to host ranged between 9.7% and 100%. *Ae. fowleri* with a disseminated infection fed on hamsters lead to 61% of these hosts being infected, and all hamsters were infected by *Ae. mcintoshi* [59]. Twenty % of *O. caspius* transmitted the virus from infected hamster to uninfected hamster. For disseminated infections this was 50% [60].

The host-to-vector-to-host transmission was determined in one experiment [62]. Of *O. caspius* feeding on hamsters, 9.7% to 23.1% transmitted the infection to an uninfected hamster [62].

Vertical transmission of the virus to *Aedes* mosquito eggs is indicated as a way for RVFV to bridge inter-epidemic periods. This idea is based on the findings in the early 1980’s of infected larvae and pupae of *Ae. lineatopennis* in Kenya [9]. Of these field collections, 2 out of 279 emerging females and 1 out of 731 emerging males were infected. This is only 0.7% of females () and 0.3% of males. Studies that reproduce these findings under laboratory conditions are unknown to our knowledge.

In summary, in study we used that the probability of transmission from host to *Aedes vexans*, followed by dissemination, is 0.38 and from *Aedes vexans* with disseminated infection to host is 0.70. The ranges are wide so we used a range of 0.0 to 1.0 in the uncertainty analysis. Vertical transmission was studied with a probability of 0.007 and a range between 0 and 0.015 ().

#### Culex pipiens s.l.

For transmission from host to vector, *Cx. pipiens* s.l. disseminated infections were observed in 18%-22% of feedings [59], and in another study 45% [15].

Transmission from *Cx. pipiens* s.l. to hamster was found in 46.2% [56] to 100% [15]. Also mechanical transmission to lambs is reported for mosquitoes feeding on viraemic hamsters, but mechanical transmission by mosquitoes is considered to play a minor role [11].

In summary, we used a host to *Culex pipiens* s.l. transmission probability of 0.22, and a *Culex* *pipiens* s.l. to host probability of 0.78 ().

### Host preference of the vector

Host preferences, as used in the model, are expressed as relative numbers, of which that for the most preferred host is set to 1.0 ().

#### Aedes vexans

Comparison of different baits in traps showed that the bovine-baited net was by far the most effective trap to catch *Aedes vexans*, with 53.6% of all collected *Ae. vexans* mosquitoes in that trap. This was followed by the sheep-baited net (16.7%), man-baited net (12.6%) and chicken-baited net (11.6%) [53]. Field collected mosquitoes in Senegal showed that overall 53.2% of the blood meals from *Ae. vexans* were taken on equine, 18.6% on bovines, 7.1% on sheep and 0.6% on human. No blood meal was taken on rodents [53]. In the United States *Ae. vexans* collected in nature had fed in 80% on mammals, consisting of humans (31%) and white tailed deer (48%) [11]. As no host densities are known in these nature areas, these figures are only indicative for a preference towards mammals, which is confirmed by others [64].

#### Culex pipiens

Determining the host preference of *Culex pipiens* s.l. is fraught with uncertainty as this vector is a complex of subspecies, which range from pure ornithophilic to totally opportunistic mosquitoes [64]. The subspecies hybridize, producing populations with intermediate preferences. For example 52% of *Cx. pipiens* s.l. caught in Egyptian villages had fed on humans, 9.8% on cattle and 1.8% on sheep. Only 4.5% had fed on chicken [65]. In the United States, 16% of *Cx. pipiens* s.l. had fed on mammals [63], and in Russia a similar 19% had fed on humans [66]. We chose a relative preference of 0.2 over birds. However, as no birds were included in the model study, we implicitly assumed that this vector species has an equal preference for cattle, sheep and goats.

## Population sizes of hosts and vectors

### Host

The abundances of hosts and vectors per 5 × 5 km grid in the Netherlands were acquired from external sources. The abundances of hosts (domestic cattle, sheep, goats) were determined from a database of “Dienst Regelingen” of the Ministry of Economic Affairs, Agriculture and Innovation (EL&I).

### Vector

The mosquito abundances, consisting of *Ae. vexans* and *Cx. pipiens* s.l., were determined by Avia-GIS. The report can be obtained through the authors [19]. In short, the mosquito abundances by Avia-GIS are based on 1000 sampling points from mosquito traps in Belgium in 2007-2008, which were then extrapolated to the Netherlands using landscape, vegetation, temperature, precipitation, distance to water and soil data.

Abundance models were made in a two-step approach. First absence-presence models were created and secondly abundance models were generated. In both steps, models were generated using Random Regression Forests. This method allows internal and external validation by bootstrapping methods. A third of the data points is left out of the analysis and used for external validation.

For the models 50 variables were included, and the probability of presence was added as an extra variable for the abundance model. In the final model, the most important variables included probability of presence, climate related variables, vegetation variables and distances to urban or protected areas, and distance to water. Population or livestock related variables were not included in the final model. The coefficient of determination was high for both species (R^2^ = 0.93 for *Ae. vexans* and R^2^ = 0.88 for *Cx.pipiens*) [19].

Mosquito abundances are assumed to be related to these properties, as that represents the availability of breeding sites, and are assumed to be independent of host densities.

The mosquito abundance is here the yearly maximum number of mosquitoes expected to be caught during a 7 day catch with CO_2_ traps. This population only consists of females, as they search for a blood meal. Following the assumptions in [36] we assumed that 1% of the total mosquito population present in an area of 1 km^2^ is caught by one trap. Multiplication with 2500 will thus result in the number of mosquitoes per 5 by 5 km grid. This crude assumption will be subject of the uncertainty analysis of the model, assuming a 10-fold smaller and larger mosquito abundance.

### Seasonality of vector abundance

The temporal changes in mosquito abundances during the year in the Netherlands were estimated from observed mosquito data of Takken et al. [67]. These data comprise of monitoring during July-October 2005 and March-July 2006 at different sites in the Netherlands for *Culex pipiens* and *Aedes cinereus*. The *Ae. cinereus* catches were used to derive the *Ae. vexans* population dynamics, because longitudinal data for that species is not available. Mosquito catches of Takken et al. [67] for which the temperature was lower than 9.6 °C (the value for which the biting rate is 0) were excluded.

CO_2_ traps catch female mosquitos searching for a blood meal, which is called aggressiveness [55]. The numbers caught by such a trap are thus the number of females which have fulfilled a gonotropic cycle. As that cycle depends on temperature (and so does the number of bites per time), the mosquito catches in CO_2_ traps depend on temperature as well. We assumed that the dynamics of the mosquito population *v(t)* could be decomposed into a Fourier series. Therefore, we started with one sinoidal pattern during the vector season with length *θ*. To derive the maximum population abundance *v_max_* and the phase *v_a_*, we used the observed catch data *C(t, T_t_)* and the biting rate *b(T_t_)* depending on temperature *T_t_* (24 h average)*.*

$\begin{matrix} C(t,T_{t})=b(T_{t})\cdot v(t) \\ \begin{matrix} \begin{matrix} & & \end{matrix} & & =b(T_{t})\cdot\frac{1}{2}v_{\max}\left(1+\sin(\frac{2\pi\cdot t-v_{a}}{\theta})\right) \end{matrix} \end{matrix}$ Equation 14

We estimated the phase *v_a_* for both populations. These were 40.72 for *Cx. pipiens* and 1.01 for *Aedes cinereus* for *t_0_* is the 20^th^ of April. These values result in a peak for *Cx. pipiens* at the beginning of September and a peak for *Aedes cinereus* at the beginning of May. The explained variance of this sinoidal population dynamics model (R^2^) was 0.42 for *Cx. pipiens* and 0.22 for *Aedes cinereus.* Introducing more Fourier components did improve the fit slightly, but because the overall pattern was not changed much, and the data were only compromised of two years, we chose to use only one Fourier component.

# Part 3: Reference curve for risk maps

A reference curve was used to create the risk maps. These risk maps visualise (based on the reference curve) the probability that the epidemic growth rate exceeds the threshold value of 1 for each grid cell of the risk map. This is an important difference with risk maps that only show the expected value of the risk indicator.

The probability of exceeding the threshold value is determined by sampling from the uncertainty distributions of the 21 input parameters. Sampling for each individual grid cell would require too much computation efforts and therefore we chose to create a reference curve, which determines the probability of exceeding the threshold as a function of the point estimate of the epidemic growth rate in the grid cell.

One hundred areas (differing in vector and host abundance) were selected randomly from the grid. For each of these one hundred areas we determined the point estimate and the probability that the threshold value would be exceeded. The probability was determined by a Latin Hypercube sampling method to provide a good coverage of the parameter space without having to do with unfeasible number of computations. The sampling method yields a distribution of epidemic growth rates for each area, of which a certain fraction is positive. We then fitted a parabolic function through the estimates which yielded the reference curve ().

This reference curve gives the probability of the exponent of the initial epidemic growth rate (*e^r^*) being larger than the threshold 1, as a function of the point estimate (i.e. the outcome of the model with the default parameter values). This reference curve was used to create risk maps showing the probability of an outbreak in each 5 by 5 km area in the country. The same reference curve was used to create maps showing this for the *R_T_*, i.e. for persistence of RVF in each 5 by 5 km area.


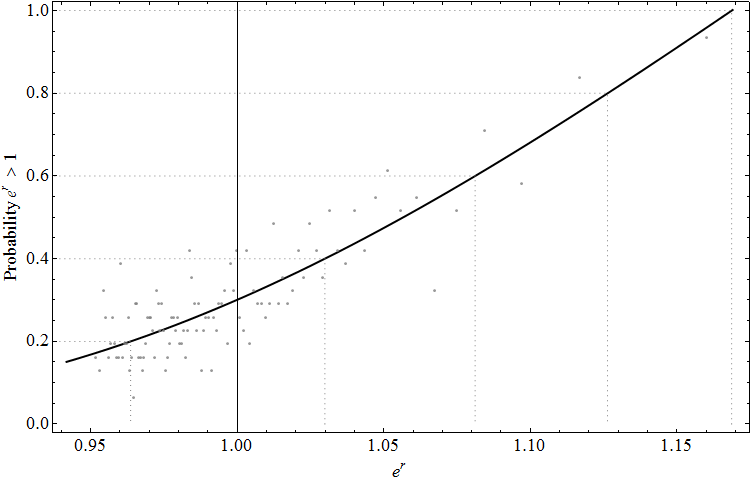


**Figure S2 Reference curve used to create the riskmaps.** The y-axis shows the probability of the exponent (*e^r^*) of the initial epidemic growth rate (at a certain time in the year) to be higher than the threshold value of 1, for different values of the point estimate of epidemic growth rate (calculated with the default parameter values) on the x-axis.

# Part 4: Direct transmission

Rift valley fever virus might be transmitted directly between animals, which increases the potential for the virus to spread, even spread in the absence of vectors. We have added transmission of the virus between all susceptible species with the same rate. This analysis shows that the log(*R­_T_*) increases linearly with direct transmission (Figure S4).


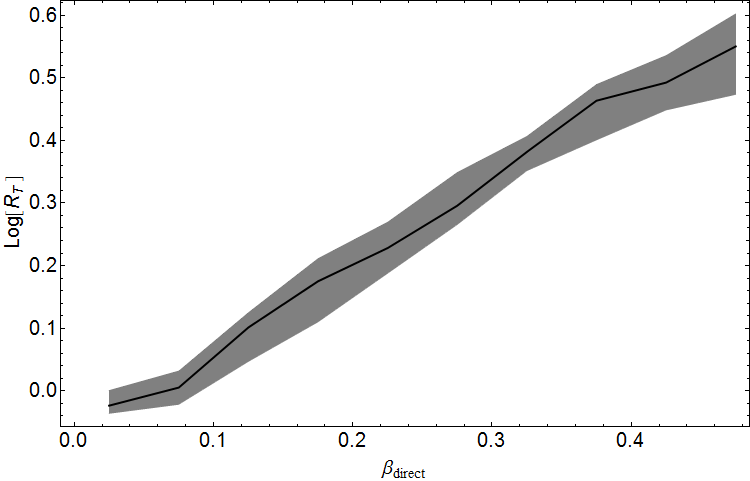


**Figure S3 Effect of direct transmission *β_direct_* on *R_T_.*** The black line is the median value of the calculation of 25 hypercube samples for each value of *β_direct_*. The grey area is the 25%-75% area of samples.

# Part 5: Sensitivity of stasis assumption

Activity and survival of mosquitoes during winter months and especially how that affects the virus, is poorly understood. Some mosquito species, including *Cx. pipiens* s.l., have strategies to survive the winter as adult [40]. Therefore, we assumed a period of stasis during winter, i.e. the number of susceptible and infected vectors and the number of susceptible, infected and recovered hosts at the beginning of the vector season is equal to the situation at the end of the previous vector season. This implies that the infection does not die out during the winter. The rationale behind this assumption is that the overwintering strategies of vectors and the virus cannot be determined for RVF in temperate countries like the Netherlands. For instance, the bluetongue virus overwintered in the country unexpectedly, and it is still not clear how. During the subsequent vector season, bluetongue detections popped up randomly in areas which had been infected before the winter (Boender et al. in prep.). This indicated that the virus had overwintered and the epidemic reactivated, when the conditions were favourable again.

For convenience we assumed stasis for the host as well, but this is unlikely to occur. Hosts will recover from the infection within a week and thus there will be no infected host at the beginning of the next favourable season. Here, we will show that assuming stasis of the host is, however, a good approximation and thus will not affect our conclusions.

Assuming stasis of the virus in host and vector during winter is equivalent to assuming that the vector activated in the beginning of the spring will infect hosts very fast, such that the ratio of infected hosts and vectors return to the same value as at the end of the previous vector season at which vector activity seized. We have tested this assumption by starting the vector season with either no infected hosts (recovery of hosts) or stasis of both vector and host (stasis). The outbreak was initiated 21 days before the end of the previous vector season.


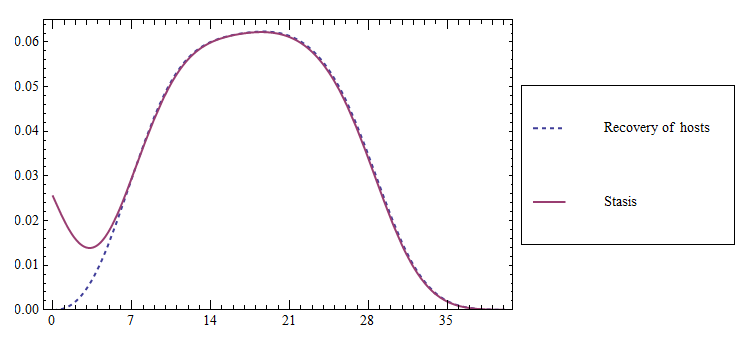


**Figure S4 Simulatons of the effect of the assumption of stasis in the first 35 weeks of the vector-season.** Y-axis depicts the ratio of infected hosts to infected vectors during thefirst 35 weeks of the vector season, when the initial values at the beginning of the vector season are either: no infected hosts (recovery of hosts) or the same number of infected hosts as at the end of the previous vector season (stasis).

Within one week after the start of the next vector season, the ratio of infected hosts to vectors has established itself at the same level in the simulations (). This means that assuming stasis of both hosts and vectors during winter does not affect the results much from assuming stasis of vectors alone.

Thus, the outcomes of the model are not sensitive to the stasis of hosts assumption and makes the calculation of the Floquet ratio *R_T_* technically easier and does not require any additional assumptions on the vector activity and survival during the winter.

**Table S1 Estimated host parameters and their range (used in the uncertainty analysis).**

| **Parameter** | **Definition** | **Value** | **Unit** | **Range** | **Ref.** |
| --- | --- | --- | --- | --- | --- |
| *1/φ^h^* | Average latent period of host | 1 | day | (0 – 2) | [4,7,47] |
| *γ^h^ * k* | Mean infectious period | 5.0 | day | (3.0 – 7.0) | [4,7,46,48] |
| *(γ^h^)^2^ * k* | Variance infectious period | 1.25 | day^2^ | (0.5 – 2.0) | [4,7,46,48] |
| *γ^h^* | Transition rate infectious classes | 0.25 | day^-1^ | (0.07 – 0.67) |  |
| *k* | Number of infectious classes | 20 | - | (5 – 98) |  |
|  |  |  |  |  |  |

**Table S2 Estimated vector parameters and their range (used in the uncertainty analysis).** Temperature dependence of parameters is modelled using the average daily (24 h) temperature of 1971-2000 observed at the centrally located meteorological institute KNMI in the Netherlands [20].

| **Parameter** | **Definition** | **Value** | **Unit** | **Range** | **Ref.** |
| --- | --- | --- | --- | --- | --- |
| *1/μ^v^_Aedes_* | Longevity of *Aedes*  *1/μ^v^_Aedes_(T) = a_0_ - a_1_ T* | *a_0_* = 25.8  *a_1_* = 0.45 | day | (11.1 – 46.8)  (-0.37 –1.67) | [50] |
| *1/μ^v^_Culex_* | Longevity of *Culex*  *1/μ^v^_Culex_(T) = a_0_ - a_1_ T* | *a_0_* = 69.1  *a_1_* = 2.14 | day | (-21.7 – 160.0)  (-1.99 – 6.28) | [51,52] |
| *d^v^_Culex_* | Increased mortality of infected *Culex* | 1.26 |  | (1.00 – 1.50) | [27] |
| *b* | Biting rate of mosquito species  *b(T) = b_slope_(T- b_min_)* | *b_min_* = 9.60  *b_slope_* = 0.0173 | day^-1^ | (7.6 – 11.6)  (0.0163 – 0.0183) | [21] |
| *1/φ^v^_Aedes_* | Extrinsic incubation period *Aedes*  *φ (T) = φ_max_- φ_slope_ T* | *φ_max_*= 18.9  *φ_slope_*= 0.30 | day | (0.0 – 37.8)  (-0.35 – 0.93) | [56-58] |
| *1/φ^v^_Culex_* | Extrinsic incubation period *Culex*  *φ (T) = φ_max_ - φ_slope_ T* | *φ_max_*= 11.3  *φ_slope_*= 0.30 | day | (0.0 – 22.6)  (-0.35 – 0.93) | [25] |

**Table S3 Estimated transmission probabilities and their range (used in the uncertainty analysis).**

| **Parameter** | **Definition** | **Value** | **Range** | **Ref.** |
| --- | --- | --- | --- | --- |
| *ζ* | Vertical transmission *Ae. vexans* | 0.007 | (0.00 – 0.015) | [9] |
| *β_Aedes_* | Host to *Aedes* | 0.38 | (0.00 – 1.00) | [59-62] |
| *β_Culex_* | Host to *Culex* | 0.22 | (0.00 – 1.00) | [15,56,59] |
| *α_Aedes_* | *Aedes* to host | 0.70 | (0.00 – 1.00) | [59,60,62] |
| *α_Culex_* | *Culex* to host | 0.78 | (0.00 – 1.00) | [15,56,59] |

**Table S4 Host preferences *π_ij_* are expressed as relative numbers, of which that for the most preferred host is set to 1.0.** For *Culex,* only data of *Cx. pipiens* s.l. are used in the model. Birds are not included as hosts in the model.

| **Vector** | **Host** | **Value** | **Range** | **Ref.** |
| --- | --- | --- | --- | --- |
| *Ae. vexans* | Cattle | 1.0 | (0.0 – 1.0) | [53,63] |
|  | Sheep and goat | 0.3 | (0.0 – 1.0) |  |
|  | Birds | 0.2 | (0.0 – 1.0) |  |
| *Cx pipiens s.l.* | Cattle | 0.2 | (0.0 – 1.0) | [63-65] |
|  | Sheep and goat | 0.2 | (0.0 – 1.0) |  |
|  | Birds | 1.0 | (0.0 – 1.0) |  |
|  |  |  |  |  |
